# Supplementary material for: A cell-type-specific alternative splicing regulator shapes synapse properties in a trans-synaptic manner
Source: Cell Rep. 2023 Mar 1;42(3):112173. doi: 10.1016/j.celrep.2023.112173 (PMC10066595; doi:10.1016/j.celrep.2023.112173)
Supplement: Document S1. Figures S1–S6 [file mmc1.pdf]

Cell Reports, Volume *42*

Supplemental information

A cell-type-specific alternative splicing regulator shapes synapse properties in a *trans*-synaptic manner

Lisa Traunmüller, Jan Schulz, Raul Ortiz, Huijuan Feng, Elisabetta Furlanis, Andrea M. Gomez, Dietmar Schreiner, Josef Bischofberger, Chaolin Zhang, and Peter Scheiffele

Supplementary Material

Figure S1 – related to Figure 1

Figure S2 – related to Figure 2

Figure S3 – related to Figure 3

Figure S4 – related to Figure 4

Figure S5 – related to Figure 4

Figure S6 - related to Figure 4

Figure S1

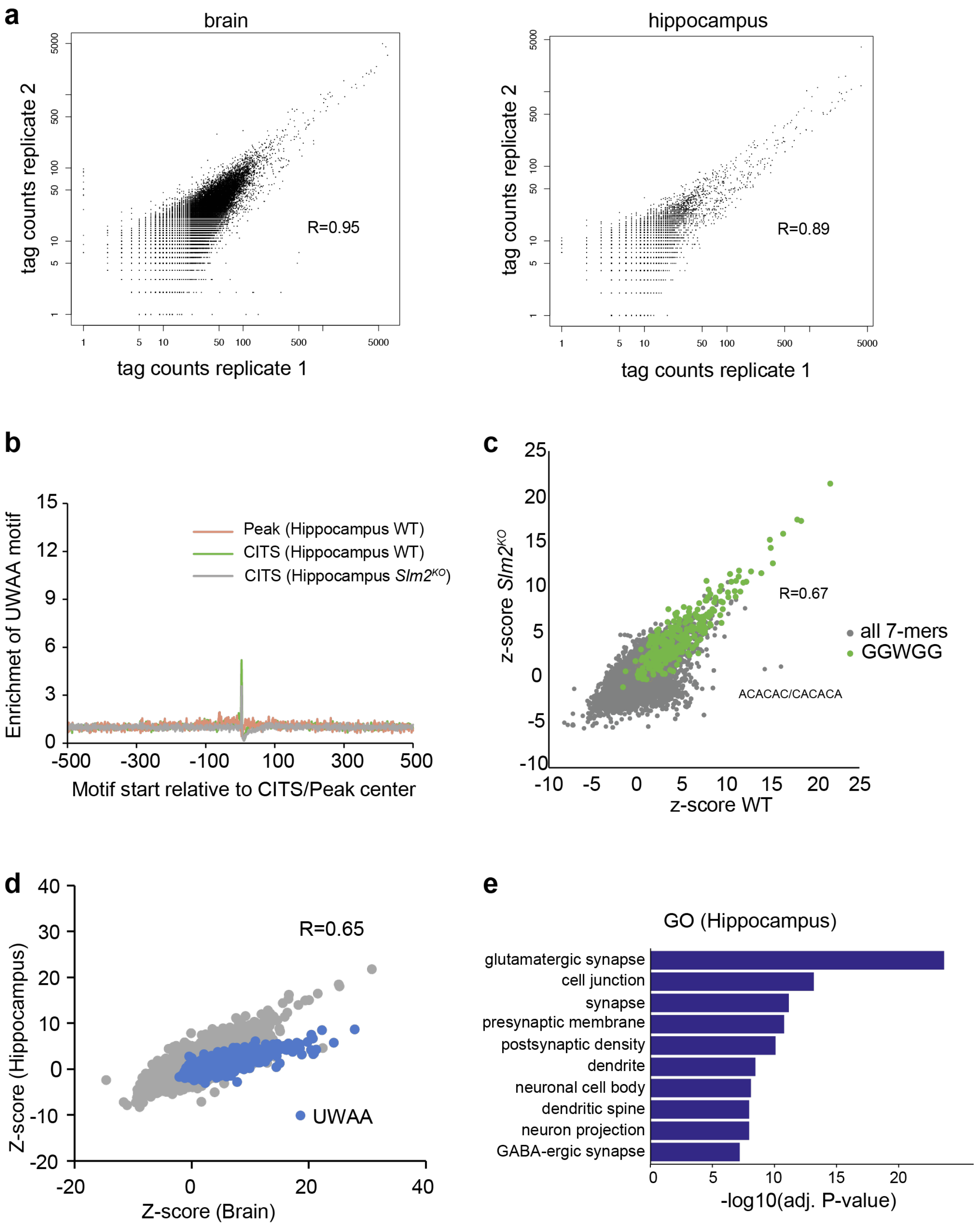
a
brain
tag counts replicate 2
tag counts replicate 1
R=0.95
hippocampus
R=0.89
b
Enrichmet of UWAA motif
Peak (Hippocampus WT)
CITS (Hippocampus WT)
CITS (Hippocampus Slm2KO)
Motif start relative to CITS/Peak center
c
z-score Slm2KO
z-score WT
R=0.67
all 7-mers
GGWGG
ACACAC/CACACA
d
Z-score (Hippocampus)
Z-score (Brain)
R=0.65
UWAA
e
GO (Hippocampus)
glutamatergic synapse
cell junction
synapse
presynaptic membrane
postsynaptic density
dendrite
neuronal cell body
dendritic spine
neuron projection
GABA-ergic synapse
-log10(adj. P-value)

Figure S1. Mapping SLM2 binding sites by eCLIP

a, Correlation plot of tag numbers over called eCLIP tag clusters in replicate 1 (x-axis) and replicate 2 (y-axis) of whole brain (left panel) and hippocampus (right panel) eCLIP data. Pearson’s correlation coefficient is shown. **b,** Enrichment of UWAA around CITS is calculated from the frequency of UWAA starting at each position relative to the inferred crosslink sites, normalized by the frequency of the element in flanking sequences in hippocampus eCLIP data from wild-type and *Slm2*^{KO} hippocampus. Enrichment of UWAA around the CLIP tag cluster peak center is shown for comparison. **c,** Correlation plot of 7mer enrichment z-scores from WT and *Slm2*^{KO} hippocampal eCLIP data. The GGWGG motif identified in hippocampal WT eCLIP samples (highlighted in green) is found to the similar extent in global SLM2 knock-out control samples. Pearson's correlation coefficient is shown. **d,** Correlation of 7-mer enrichment z-scores of 100nt region around peak center from whole brain (x-axis) and hippocampus (y-axis) eCLIP data. 7-mers including UWAA are highlighted in blue. Pearson's correlation coefficient is shown. **e,** Gene Ontology analysis (DAVID tools) of genes with SLM2 binding sites in hippocampal eCLIP data identified by CLIPper/IDR. Top 10 enriched gene ontology categories for cellular compartment are displayed.

Figure S2

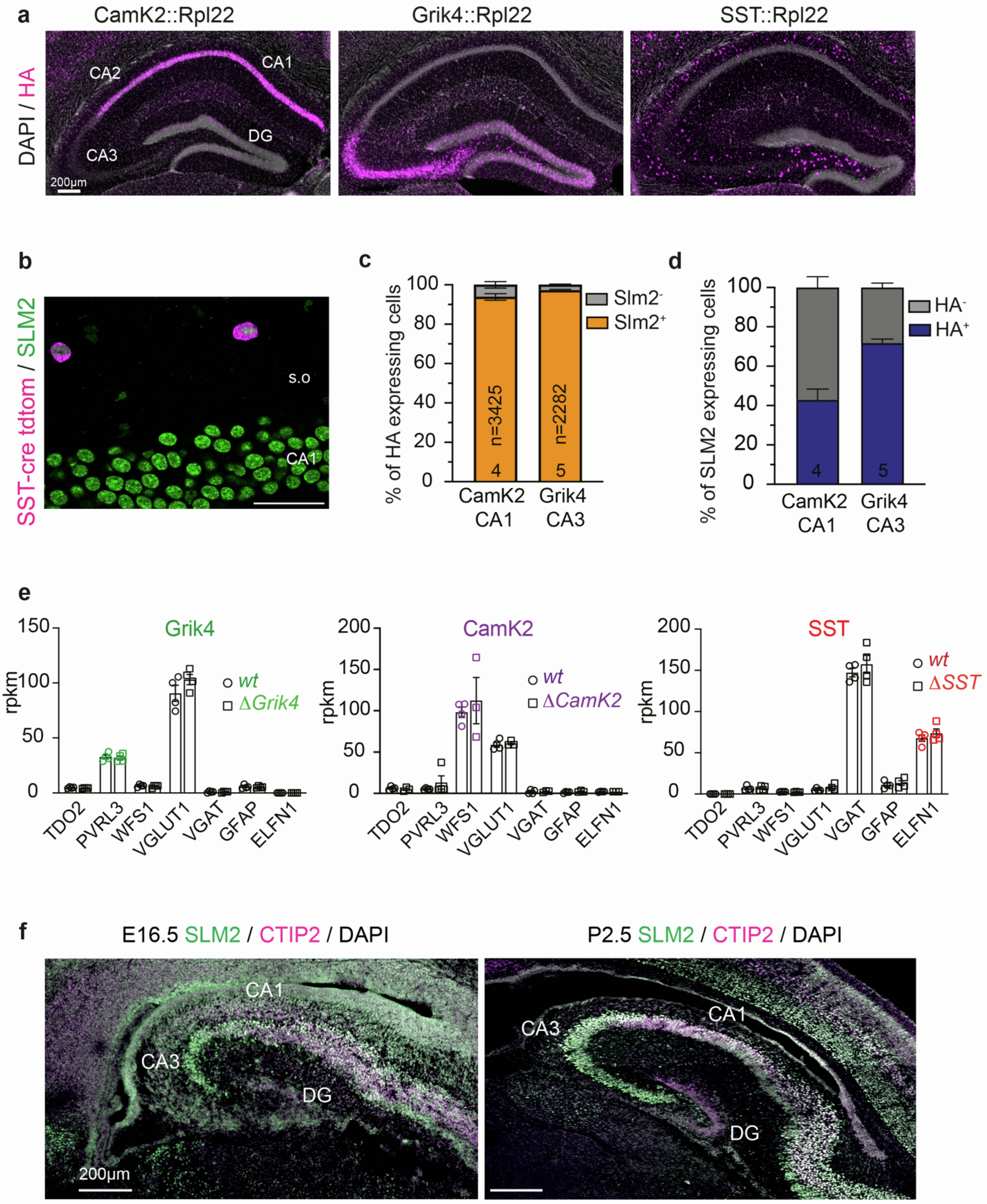
a
CamK2::Rpl22
Grik4::Rpl22
SST::Rpl22
DAPI / HA
CA2
CA1
DG
CA3
200µm
b
SST-cre tdtom / SLM2
s.o
CA1
c
% of HA expressing cells
Slm2-
Slm2+
n=3425
n=2282
CamK2 CA1
Grik4 CA3
d
% of SLM2 expressing cells
HA-
HA+
e
Grik4
CamK2
SST
rpkm
wt
ΔGrik4
ΔCamK2
ΔSST
TDO2
PVRL3
WFS1
VGLUT1
VGAT
GFAP
ELFN1
f
E16.5 SLM2 / CTIP2 / DAPI
P2.5 SLM2 / CTIP2 / DAPI
CA1
CA3
DG
200µm

Figure S2. Expression and conditional knock-out of SLM2 in the mouse hippocampus.

a, Representative images of cre-dependent expression of HA-tagged ribosomal protein L2 (Rpl22) in CA1 (CamK2::Rpl22), CA3 (Grik4::Rpl22) or SST+ interneurons (SST::Rpl22). Scale bar 200µm, DAPI (grey), HA (magenta). **b,** SLM2 (green) expression in CA1 pyramidal neurons and genetically marked SST+ interneurons (magenta) in the stratum oriens (s.o). Scale bar 40µm. **c,** Quantification of percentages of HA+ neurons defined by either CamK2 or Grik4 cre-recombinase which express SLM2 (SLM2+, orange). CamK2-cre: N=4 animals, n=3425 cells. Grik4-cre: N=5 animals, n=2282 cells. Mean of each replicate ± SEM. **d,** Quantification of SLM2+ neurons in either CA1 or CA3 layers which express Rpl22-HA (HA+, blue). Same images and numbers as for (c). Mean of each replicate ± SEM. **e,** Reads per kilobase million (rpkm) of cell class-specific marker genes in all analyzed cell types and individual replicates of *wt* and ∆*SLM2* animals (N=4). *TDO2*: DG marker, *PVRL3*: CA3, *RGS12*: CA2, *WFS1*: CA1, *VGLUT1*: excitatory neurons, *VGAT*: inhibitory neurons, *GFAP*: glia, *ELFN1*: SST neurons. **f,** Hippocampal tissue from mice immunostained for SLM2 (green), CTIP2 (purple) and DAPI (grey) at E16.5 and P2.5. This demonstrates selective expression of SLM2 in CA1 and CA3 but not dentate granule cells at early stages of hippocampal development. Scale bar is 200µm.

Figure S3

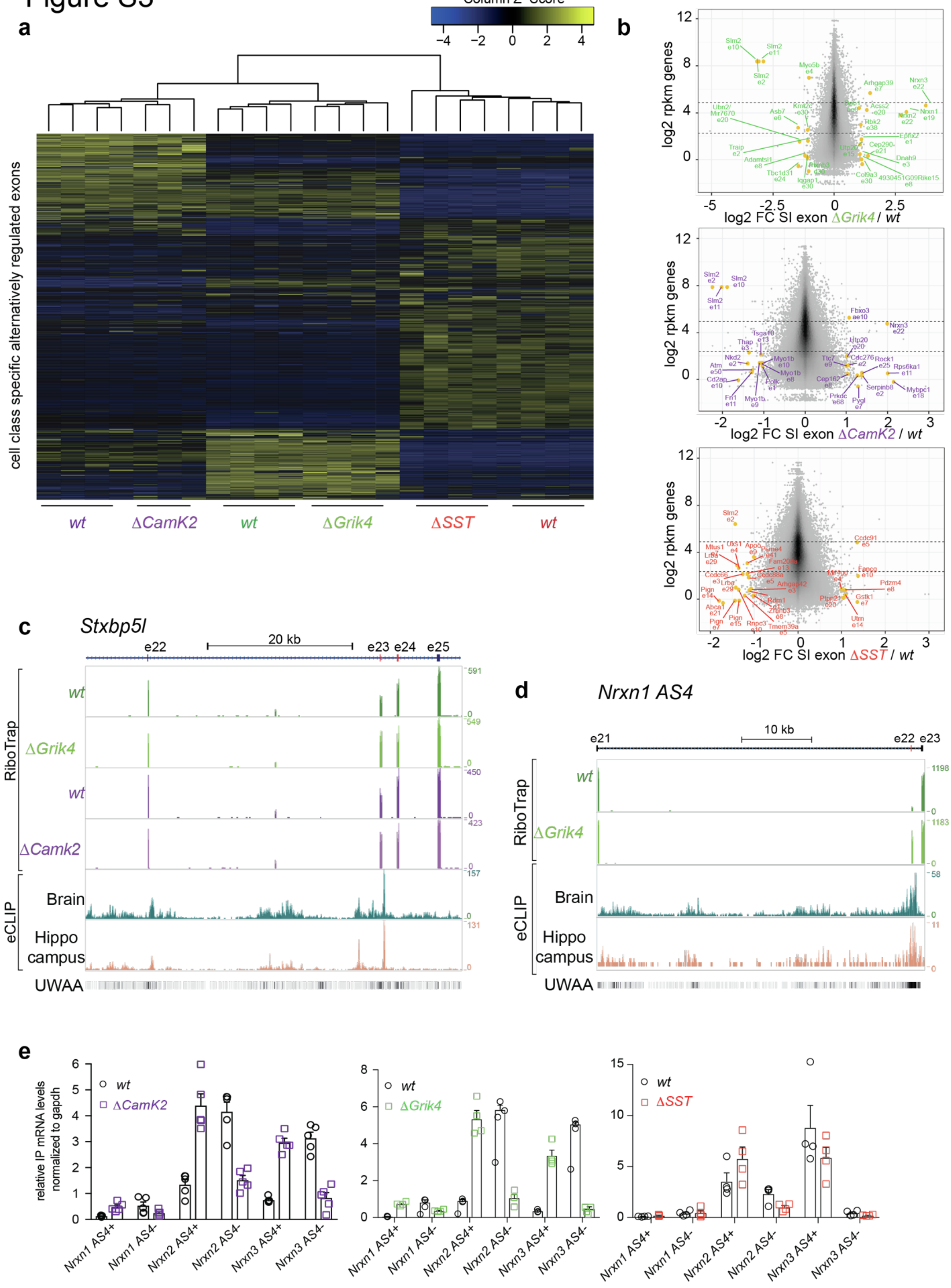
a
Column Z−Score
−4 −2 0 2 4
cell class specific alternatively regulated exons
wt
ΔCamK2
wt
ΔGrik4
ΔSST
wt
b
log2 rpkm genes
log2 FC SI exon ΔGrik4 / wt
log2 FC SI exon ΔCamK2 / wt
log2 FC SI exon ΔSST / wt
c
Stxbp5l
20 kb
e22
e23 e24 e25
RiboTrap
wt
ΔGrik4
wt
ΔCamk2
eCLIP
Brain
Hippo campus
UWAA
d
Nrxn1 AS4
10 kb
e21
e22 e23
RiboTrap
wt
ΔGrik4
eCLIP
Brain
Hippo campus
UWAA
e
relative IP mRNA levels normalized to gapdh
wt
ΔCamK2
ΔGrik4
ΔSST
Nrxn1 AS4+
Nrxn1 AS4-
Nrxn2 AS4+
Nrxn2 AS4-
Nrxn3 AS4+
Nrxn3 AS4-

Figure S3. Alternative splicing in *Slm2* conditional knock-out cells.

a, Heatmap of splicing indices (SI) of exons across individual replicates of *wt* and *Slm2* conditional mutants. Analyzed exons were defined by previously identified, cell class-specific alternative exons[1]. Splicing indices were normalized by row and column. Detailed z-score values, gene and exon names are provided in Table S2. **b,** Log2 fold change SI (splicing index) and p-values for all detected exons (grey). Exons which are significantly differentially regulated by SLM2 in each cell class (called by exon analysis) are marked in yellow. **b,** Log2 fold change of SI of all detected exons (grey) and log2 rpkm values of the corresponding gene. Differentially regulated exons called by the exon analysis are marked in yellow. Gene names and exons involved in the splicing regulation are indicated in purple for differential changes in CamK2, green for Grik4 and red for SST. **c,d,** Integration of RiboTrap and eCLIP analysis for significantly de-regulated exons of *Stxbp5l* (c) and *Nrxn1* (d). SLM2 binding sites in the downstream introns and enrichment of the UWAA binding motif of the Grik4 comparison are illustrated. **e,** Quantitative PCR for alterations in *Nrxn* splicing at the alternatively spliced segment 4 (AS4). Relative *Gapdh* normalized mRNA levels of RiboTRAP IP samples in WT and ΔSLM2 samples. For all PCRs: *wt* CamK2 and Δ*CamK2*: N=5; *wt* Grik4 N=4 and Δ*Grik4* N=5, *wt* SST N=4 and Δ*SST* N=4, except for *Nrxn1*$^{AS4+}$ N=3. *wt* CamK2 vs Δ*CamK2: Nrxn1*$^{AS4-}$ p= 0.861, *Nrxn1*$^{AS4+}$ p=0.0019, *Nrxn2*$^{AS4-}$ p=0.0002, *Nrxn 2*$^{AS4+}$ p=0.0003, *Nrxn3*$^{AS4-}$ p<*0.0001, Nrxn 3*$^{AS4+}$ p<0.0001; *wt* Grik4 vs Δ*Grik4 Nrxn1*$^{AS4-}$ p=0.0035, *Nrxn1*$^{AS4+}$ p<0.0001, *Nrxn2*$^{AS4-}$ p<0.0001, *Nrxn2*$^{AS4+}$ p<0.0001, *Nrxn3*$^{AS4-}$ p<0.0001, *Nrxn3*$^{AS4+}$ p<0.0001, *wt* SST vs Δ*SST Nrxn2*$^{AS4-}$ p=0.0251.

Figure S4

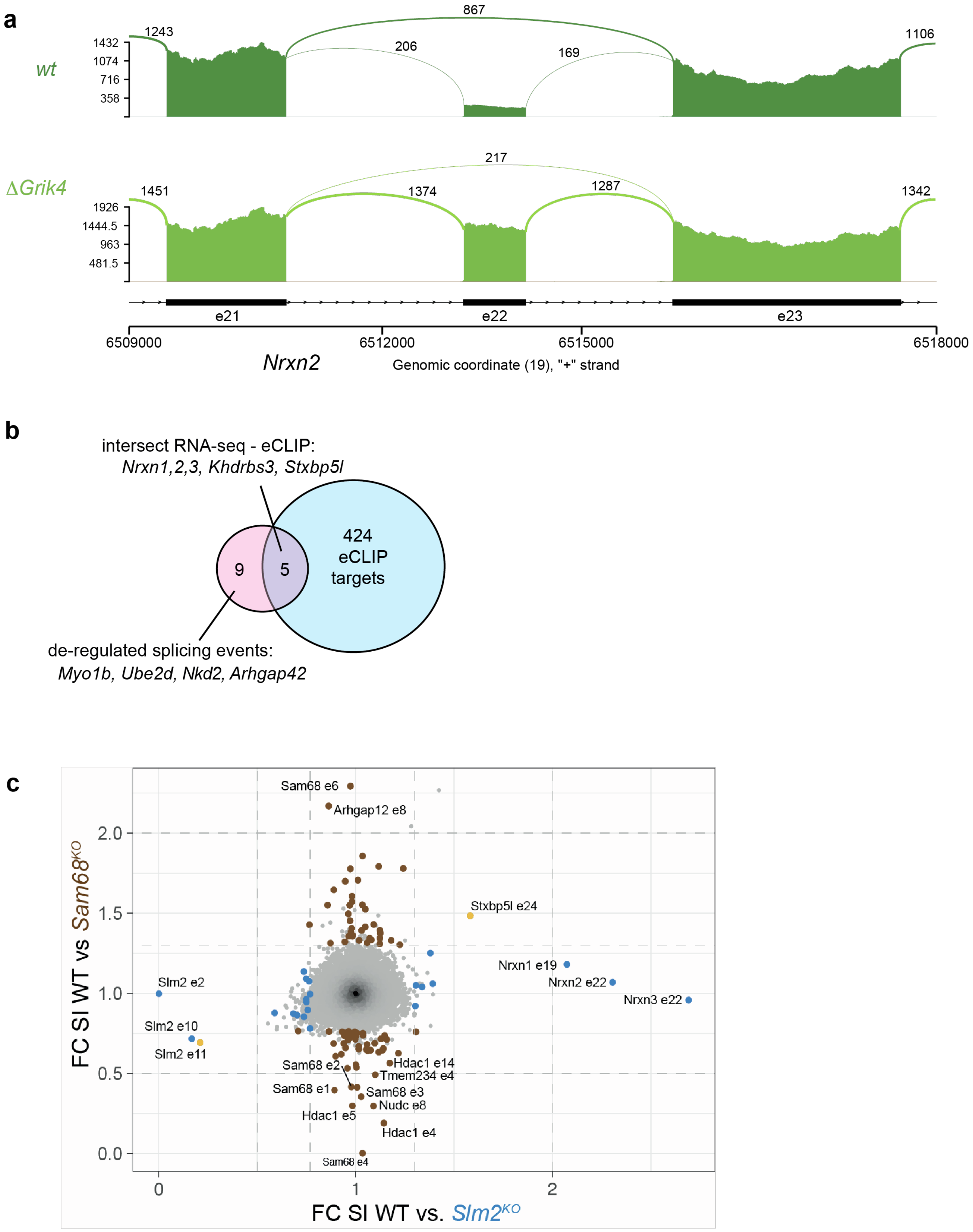
a
wt
1432
1074
716
358
1243
867
206
169
1106
ΔGrik4
1926
1444.5
963
481.5
1451
217
1374
1287
1342
e21
e22
e23
6509000
6512000
6515000
6518000
Nrxn2
Genomic coordinate (19), "+" strand
b
intersect RNA-seq - eCLIP:
Nrxn1,2,3, Khdrbs3, Stxbp5l
9
5
424
eCLIP
targets
de-regulated splicing events:
Myo1b, Ube2d, Nkd2, Arhgap42
c
Sam68 e6
Arhgap12 e8
Stxbp5l e24
Nrxn1 e19
Nrxn2 e22
Nrxn3 e22
Slm2 e2
Slm2 e10
Slm2 e11
Sam68 e2
Hdac1 e14
Tmem234 e4
Sam68 e1
Sam68 e3
Nudc e8
Hdac1 e5
Hdac1 e4
Sam68 e4
2.0
1.5
1.0
0.5
0.0
0
1
2
FC SI WT vs Sam68KO
FC SI WT vs. Slm2KO

Figure S4. Alternative splice targets in *Slm2* conditional knock-out cells.

a, Representative sashimi plots illustrating read distribution and splice junctions on the *Nrxn2* gene at *AS4* for *wt* and Δ*Grik4* conditional mutant. Genomic coordinates and exon numbers are indicated below. Junction reads for exon-exon boundaries are noted and illustrated by line thickness. **b,** Venn diagram demonstrating the number of genes identified by eCLIP/IDR as bound (424), differentially alternatively spliced in *Slm2*^{KO} (9) or both bound and alternatively spliced (5). **c,** Correlation plot of the splicing index fold change (FC SI) in mouse hippocampus between WT and *Slm2* global knock-out (*Slm2*^{KO} [2]) and WT and Sam68 global knock-out (*Sam68*^{KO} [3]) for all detected exons (grey). Significantly differentially regulated exons (FC $\pm$ 30%, p-value 0.01) are marked in brown for *Sam68* and blue for *Slm2* mutants. Two exons, marked in yellow, are commonly de-regulated suggesting very little overlap in splicing regulation by SAM68 and SLM2 proteins.

Figure S5

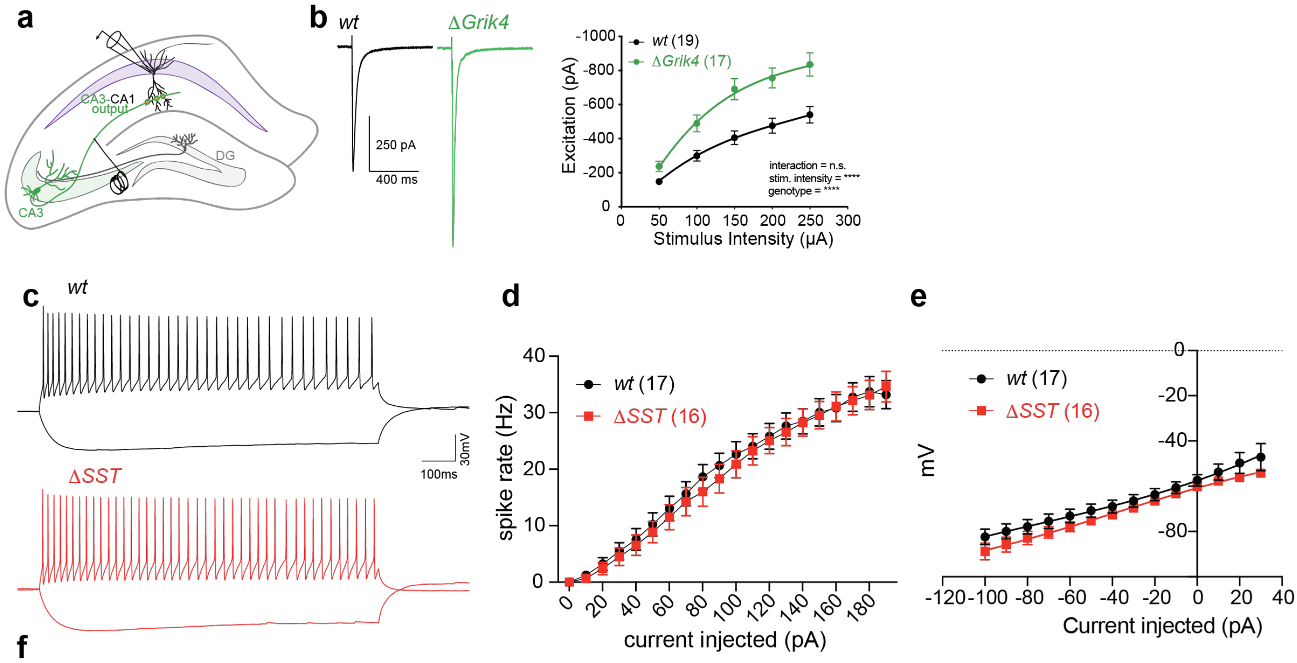

f

	WT	Slm2$^{\Delta SST}$	p-value	n (cells)
IR (MΩ)	622.9 ± 52.92	603.0 ± 46.64	0.7778	28 / 32
Capacitance (pF)	95.07 ± 7.498	101.0 ± 5.103	0.1116	28 / 32
RMP (mV)	-59.29 ± 1.964	-61.04 ± 1.250	0.4616	14 / 15
AP threshold (mV)	-38.31 ± 1.297	-38.57 ± 1.581	0.9027	14 / 15
AP amplitude (mV)	90.53 ± 3.277	93.41 ± 2.809	0.7130	14 / 15
AP Latency (ms)	83.86 ± 7.289	84.05 ± 5.358	0.4864	15 / 15
AP slope rise	283.9 ± 18.11	276.6 ± 14.64	0.4363	15 / 15
AP slope decay	116.0 ± 9.199	109.9 ± 6.326	0.2496	15 / 15
Rheobase (pA)	35.76 ± 7.923	42.27 ± 5.828	0.1612	17 / 15
Sag (mV)	-8.903 ± 1.817	-8.617 ± 1.084	0.8934	18 / 15

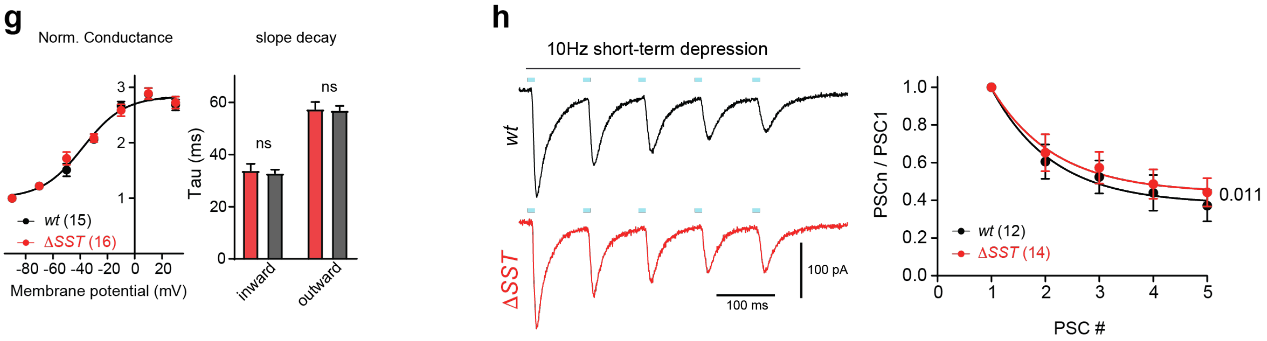

Figure S5. Electrophysiological analysis of *Slm2* conditional knock-out cells.

a, Experimental design for electrical stimulation of Schaffer collaterals and voltage clamp recordings in CA1 pyramidal cells in *wt* and ∆*Grik4* mutants. **b,** Representative traces of post-synaptic EPSCs *wt* (black) and ∆*Grik4* mutants (green). Electrically evoked EPSCs with various stimulation intensities in *wt* (n=19) and ∆*Grik4* mutants (n=17). Mean SD is displayed, two-way ANOVA was used for statistical analysis. **c,** Representative current clamp recordings to measure spike frequency of *wt* (black) and ∆*SST* (red) SST+ interneurons in s.o. Responses to a single 1s long -100pA or +150pA current injection. **d,** Frequency of action potential firing in response to increasing current injections. *wt* n= 17, ∆*SST* n=16 **e,** Analysis of changes in membrane potentials with increasing current in pA. The resting membrane potential is displayed at 0pA injection. *wt* n= 17, ∆*SST* n=16 **f,** Summary table of intrinsic electrophysiological properties of *wt* and ∆*SST* neurons. Mean ± SEM, p-values were determined by the corresponding t-tests based on assessment of normality distribution and standard deviation (see methods for details). **g,** left, a plot of normalized conductance versus membrane potential shows a clear voltage-dependence *wt* and ∆*SST* mutants. There was no genotype-dependent difference (p=0.60, Extra sum-of-squares F test). Right, analysis of decay times, weighted tau in milliseconds, of inward (at -90mV) and outward (at -10mV) currents showed no difference between genotypes (*wt* n=16 and ∆*SST* n=15) **h,** Example traces of IPSCs during repetitive stimulation at 10Hz, *wt* (black) and ∆*SST* (red). Group data of IPSCs normalized to the first peak. Mean ± SEM, *wt* n=14 and ∆*SST* n=12, Extra sum-of-squares F test for comparison of independent fits.

Figure S6

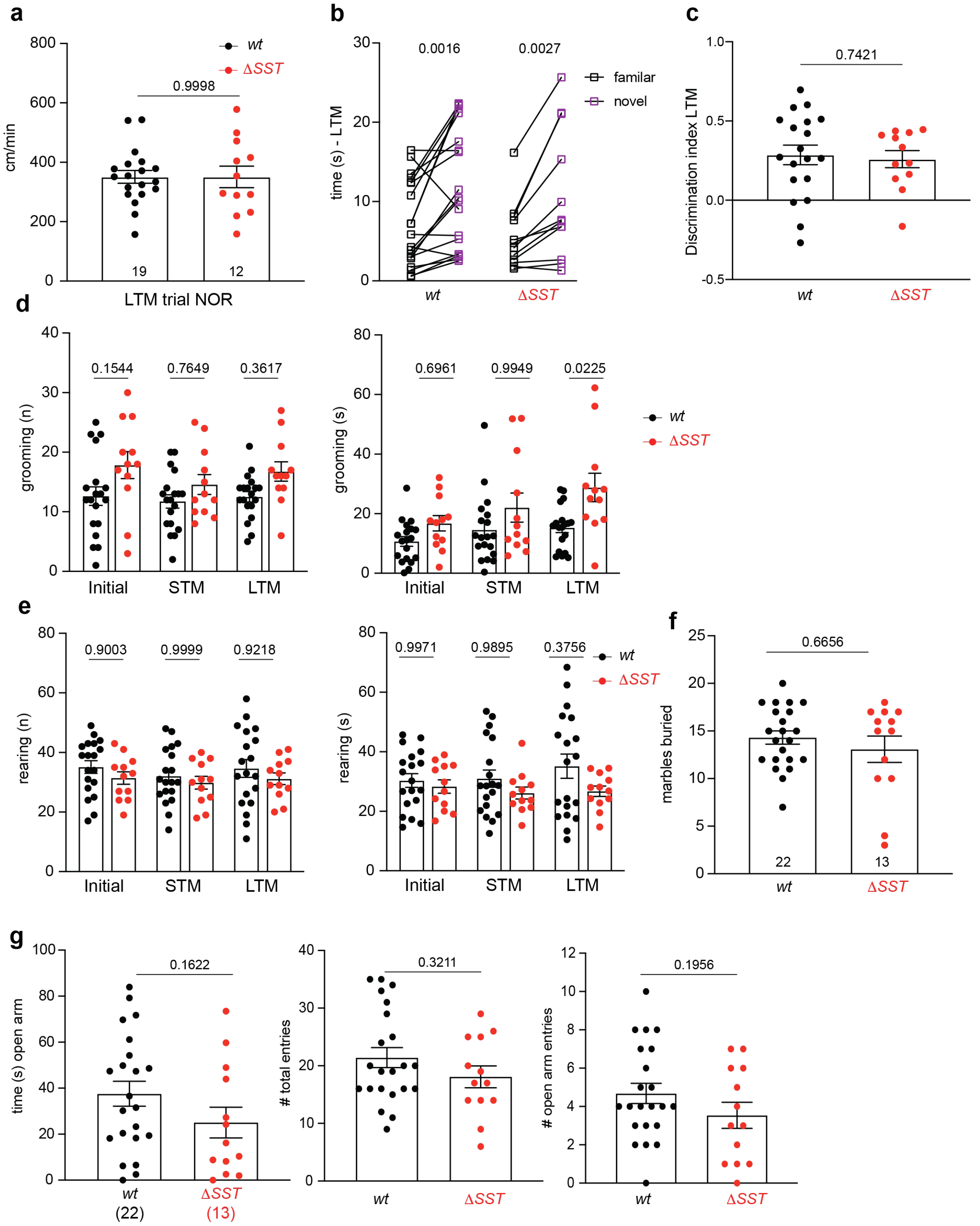
a
b
c
wt
ΔSST
0.9998
cm/min
19
12
LTM trial NOR
0.0016
0.0027
time (s) - LTM
familar
novel
0.7421
Discrimination index LTM
d
0.1544
0.7649
0.3617
grooming (n)
Initial
STM
LTM
0.6961
0.9949
0.0225
grooming (s)
e
0.9003
0.9999
0.9218
rearing (n)
0.9971
0.9895
0.3756
rearing (s)
f
0.6656
marbles buried
22
13
g
0.1622
time (s) open arm
(22)
(13)
0.3211
total entries
0.1956
open arm entries

Figure S6. Behavioral assessments in *Slm2$^{\Delta SST}$* mice

a, Quantification of velocity (cm/min) of mice during the long-term memory (LTM) test phases of the novel object recognition (NOR) task. Animal numbers for each task are indicated, Mean ± SEM, Unpaired t-test. **b,c**, Interaction time (in seconds) that mice spend with either a familiar (black) or novel (purple) object during a 5-min long-term memory trial (paired t-test) and discrimination index (unpaired t-test with Welch’s correction) are displayed. Mean ± SEM, *wt* n=19 and Δ*SST* n=12. **d,** Quantification of the number (left) and duration (right) grooming events of mice during the Open Field and phases of the NOR task. Mean ± SEM, One-Way ANOVA with Tukey's multiple comparisons test. **e,** Quantification of the number (left) and duration (right) rearing events of mice during the Open Field and phases of the NOR task. Mean ± SEM, One-Way ANOVA with Tukey's multiple comparisons test. **f,** Number of marbles buried when mice are placed in a novel homecage including 20 black marbles for 30min. Mean ± SEM, Mann Whitney t-test. *wt* n=22 and Δ*SST* n=13. **g**, Analysis of the amount of time mice spend in an open arm of the elevated plus maze during a 5min trial (left), their number of entries into either the open or closed arm (middle) and number of entries into the open arm (right). Mean ± SEM, unpaired t-test. *wt* n=22 and Δ*SST* n=13.

References Cited in Supplement

1. Furlanis, E., Traunmuller, L., Fucile, G., and Scheiffele, P. (2019). Landscape of ribosome-engaged transcript isoforms reveals extensive neuronal-cell-class-specific alternative splicing programs. Nature neuroscience *22*, 1709-1717. 10.1038/s41593-019-0465-5.
2. Traunmüller, L., Gomez, A.M., Nguyen, T.-M., and Scheiffele, P. (2016). Control of neuronal synapse specification by highly dedicated alternative splicing program. Science (New York, N.Y *352*, 982-986.
3. Witte, H., Schreiner, D., and Scheiffele, P. (2019). A Sam68-dependent alternative splicing program shapes postsynaptic protein complexes. Eur J Neurosci *49*, 1436-1453. 10.1111/ejn.14332.
