# Supplementary material for: A cell-type-specific alternative splicing regulator shapes synapse properties in a trans-synaptic manner
Source: Cell Rep. 2023 Mar 1;42(3):112173. doi: 10.1016/j.celrep.2023.112173 (PMC10066595; doi:10.1016/j.celrep.2023.112173)
Supplement: Document S2. Article plus supplemental information [file mmc5.pdf]

Cell Reports

Report

A cell-type-specific alternative splicing regulator shapes synapse properties in a *trans*-synaptic manner

Graphical abstract

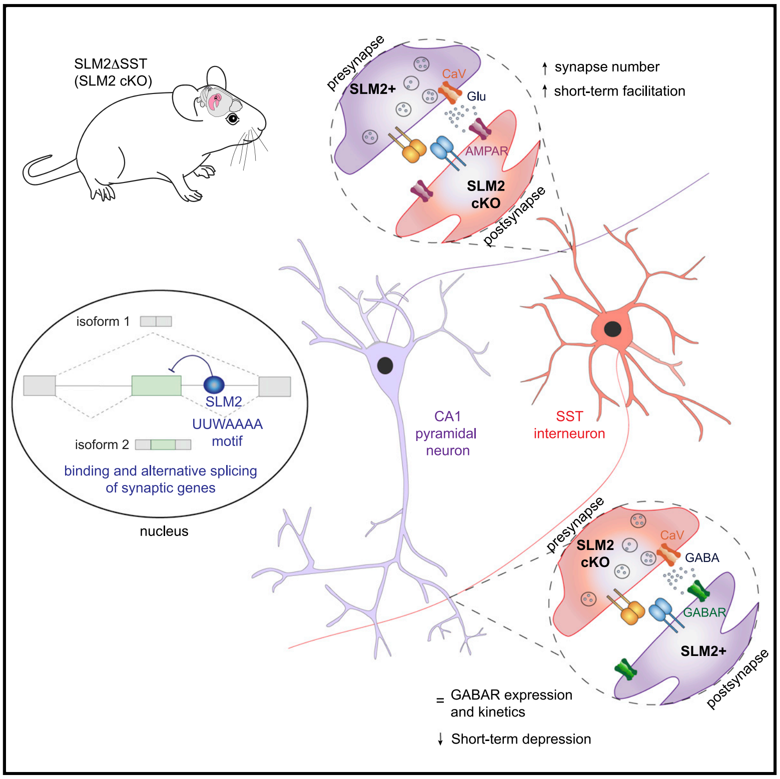

Authors

Lisa Traunmüller, Jan Schulz, Raul Ortiz, ..., Josef Bischofberger, Chaolin Zhang, Peter Scheiffele

Correspondence

peter.scheiffele@unibas.ch

In brief

Traunmüller et al. perform a systematic genetic dissection of cell-type-specific function of the alternative splicing regulator SLM2 in the mouse hippocampus. They show that SLM2-dependent alternative splicing specifically instructs the specification of synaptic properties in a *trans*-synaptic manner.

Highlights

- The alternative splicing factor SLM2 binds mRNAs encoding synaptic proteins
- SLM2 regulates splice isoforms in a cell-type-specific manner
- SLM2 loss results in selective non-cell-autonomous alterations in synaptic function
- Mice lacking SLM2 in somatostatin interneurons exhibit short-term memory deficits

Traunmüller et al., 2023, Cell Reports *42*, 112173
March 28, 2023 © 2023 The Authors.
https://doi.org/10.1016/j.celrep.2023.112173

Cell Reports

CellPress OPEN ACCESS

Report

A cell-type-specific alternative splicing regulator shapes synapse properties in a *trans*-synaptic manner

Lisa Traunmüller,[1,4] Jan Schulz,[2,6] Raul Ortiz,[1] Huijuan Feng,[3] Elisabetta Furlanis,[1,4] Andrea M. Gomez,[1,5] Dietmar Schreiner,[1] Josef Bischofberger,[2] Chaolin Zhang,[3] and Peter Scheiffele[1,7,*]

[1]Biozentrum of the University of Basel, 4056 Basel, Switzerland
[2]Department of Biomedicine, University of Basel, 4056 Basel, Switzerland
[3]Department of Systems Biology, Department of Biochemistry and Molecular Biophysics, Center for Motor Neuron Biology and Disease, Columbia University, New York, NY 10032, USA
[4]Present address: Harvard Medical School, Boston, MA 02115, USA
[5]Present address: University of California, Berkeley, Berkeley, CA 94720, USA
[6]Present address: Neuroscience & Rare Disease (NRD) Discovery and Translational Area, Roche Pharmaceutical Research and Early Development, Roche Innovation Center Basel, 4070 Basel, Switzerland
[7]Lead contact
*Correspondence: peter.scheiffele@unibas.ch
https://doi.org/10.1016/j.celrep.2023.112173

SUMMARY

The specification of synaptic properties is fundamental for the function of neuronal circuits. "Terminal selector" transcription factors coordinate terminal gene batteries that specify cell-type-specific properties. Moreover, pan-neuronal splicing regulators have been implicated in directing neuronal differentiation. However, the cellular logic of how splicing regulators instruct specific synaptic properties remains poorly understood. Here, we combine genome-wide mapping of mRNA targets and cell-type-specific loss-of-function studies to uncover the contribution of the RNA-binding protein SLM2 to hippocampal synapse specification. Focusing on pyramidal cells and somatostatin (SST)-positive GABAergic interneurons, we find that SLM2 preferentially binds and regulates alternative splicing of transcripts encoding synaptic proteins. In the absence of SLM2, neuronal populations exhibit normal intrinsic properties, but there are non-cell-autonomous synaptic phenotypes and associated defects in a hippocampus-dependent memory task. Thus, alternative splicing provides a critical layer of gene regulation that instructs specification of neuronal connectivity in a *trans*-synaptic manner.

INTRODUCTION

Neuronal synapses are small but remarkably specialized cell-cell contacts. Across synapses, their strength, the probability of neurotransmitter release, and plasticity properties are tightly controlled and represent the basis for neuronal computations. While individual neuronal cells exhibit reproducible intrinsic properties that are linked to the genetic cell identity, the synaptic properties are a function of both the pre- and postsynaptic partner cell. Thus, a single neuron can form synapses with dramatically different functional properties on two different target cell types.[1–3] The genetic mechanisms underlying the specification of these properties are incompletely understood.

Pre- and postsynaptic compartments encompass a high concentration of specific protein complexes that coalesce around nascent cell contacts. One candidate mechanism for generating target-specific synapse properties are *trans*-synaptic recognition codes that recruit select ion channels and neurotransmitter receptors in the opposing synaptic membrane.[4–11] Posttranscriptional mechanisms such as regulated alternative splicing are hypothesized to play a critical role in this process.[12–14] Cross-species comparisons demonstrated a significant expansion of alternative exon usage in organisms and tissues with high phenotypic complexity. Thus, alternative splicing programs are particularly complex in the nervous system and have vastly expanded in mammals and primates.[15–17] Moreover, the high degree of splicing regulation in the brain is accompanied by the expression of a large number of neuronal splicing regulators.[18] Recent rodent studies mapped developmental and cell-type-specific alternative splicing programs in neurons.[14,19–24] The targets of such regulation are enriched for risk genes associated with neurodevelopmental disorders,[19,25] and alterations in splicing events are associated with autism spectrum disorders in the human population.[26–28]

Genetic deletion of pan-neuronal RNA-binding proteins results in severe alterations in vast programs of alternative splicing, and simultaneous deletion of multiple RNA-binding protein (RBP) paralogs often results in embryonic or perinatal lethality.[25,29–33]

This is an open access article under the CC BY license (http://creativecommons.org/licenses/by/4.0/).

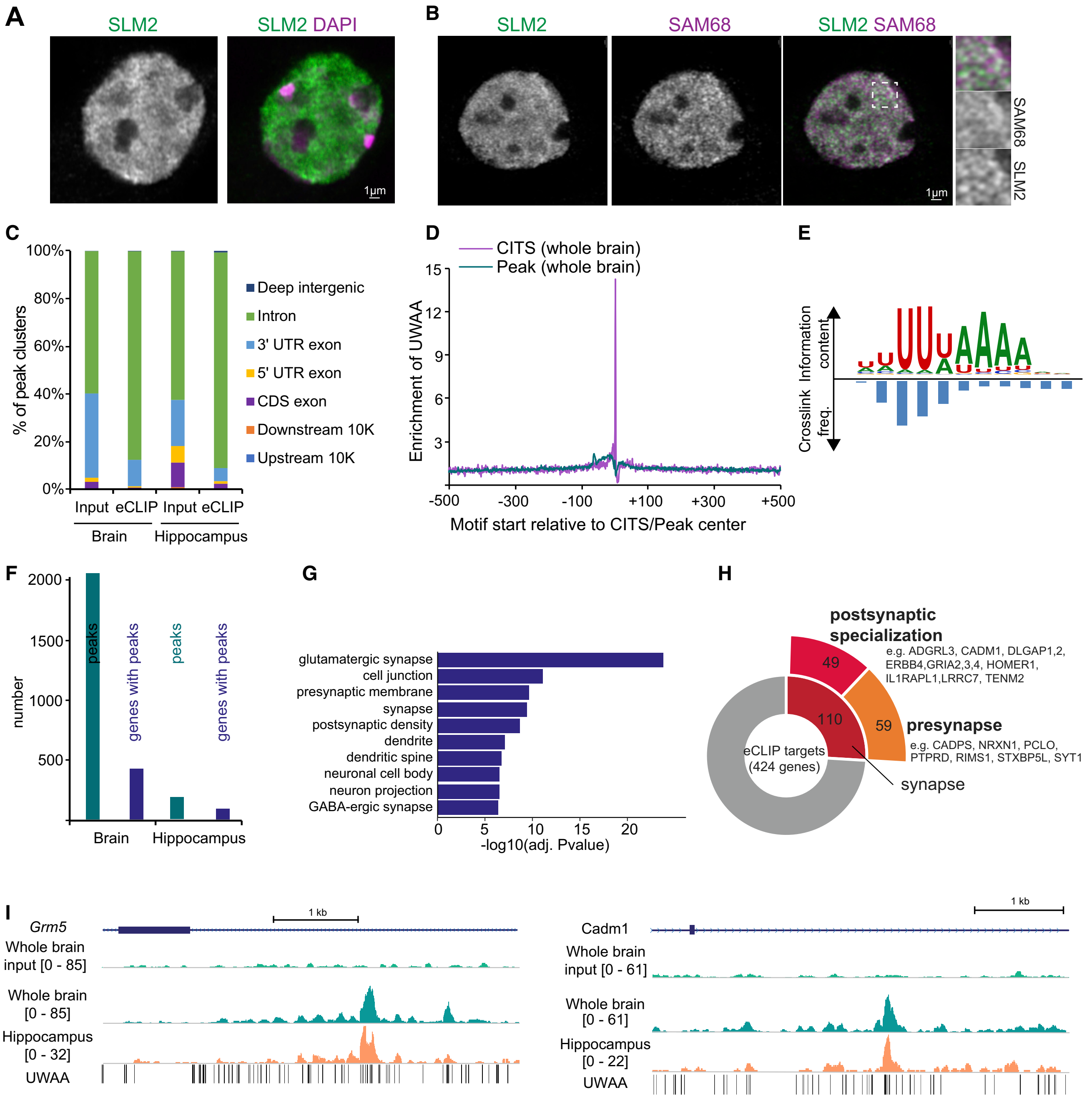

Figure 1. SLM2-bound mRNAs encode synaptic proteins

(A) Endogenous SLM2 immunoreactivity (green) and DAPI (purple) in hippocampus. Scale bar, 1 μm.

(B) Immunostaining for SLM2 (green) and SAM68 (magenta) in cultured hippocampal neurons (day *in vitro* 12). Insets show enlargement of boxed area. Scale bar, 1 μm.

(C) Cluster annotation summary for input and eCLIP samples.

(D) Enrichment of UWAA around cross-link-induced truncation site (CITS), calculated from frequency of UWAA starting at each position relative to the inferred cross-link sites, normalized by frequency of the element in flanking sequences in whole-brain eCLIP data. Enrichment of UWAA around the CLIP tag cluster peak center shown for comparison.

(E) SLM2 binding motif determined by mCross in whole-brain eCLIP data. Cross-linking frequencies at each motif position represented by blue bars.

(F) Number of high-confidence SLM2 targets identified by CLIPper and IDR analysis ($\log_2$ fold change $\geq$ 2 and $-\log_{10}$(IDR) $\geq$ 2) in whole-brain and hippocampal eCLIP samples.

(G) Gene Ontology analysis (DAVID tools) of genes with significant SLM2 binding sites (whole brain). Top 10 enriched Gene Ontology categories for cellular compartment displayed.

(legend continued on next page)

These studies firmly established a critical role for alternative splicing regulators in neural development. However, it has been difficult to dissociate specific functions of RBPs in controlling synaptic connectivity and function from a more general requirement for cell specification and viability. The KH-domain-containing paralogs SLM1 and SLM2 exhibit highly selective expression in neuronal cell types, raising the possibility that they may contribute to the terminal differentiation of these cells.[34–36] Global genetic ablation of SLM2 results in increased synaptic transmission, loss of long-term potentiation at Schaffer collateral synapses in the hippocampus, and altered animal behavior.[37,38] However, the molecular logic of how these neuronal cell-type-specific splicing regulators contribute to the acquisition of synaptic properties remains largely unclear.

Here, we systematically probed the function of SLM2, which is highly expressed in glutamatergic CA1 and CA3 pyramidal cells and a sub-set of somatostatin-positive GABAergic interneurons in the mouse hippocampus.[35,39] We combined genome-wide mapping of SLM2-bound mRNAs *in vivo* with conditional loss-of-function analyses in hippocampal pyramidal cells and somatostatin (SST) interneurons in the *stratum oriens* of hippocampus area CA1. We find that SLM2 selectively controls alternative splicing of synaptic proteins, as well as synaptic function and plasticity in a *trans*-synaptic manner. We propose that cell-type-specific alternative splicing regulators like SLM2 provide a key mechanism for instructing the molecular identity of synaptic interaction modules in mammals.

RESULTS

SLM2-bound mRNAs encode synaptic proteins

In the mouse hippocampus, SLM2 is expressed in glutamatergic pyramidal cells but also a sub-population of GABAergic interneurons.[34–36] These include *oriens-alveus lacunosum-moleculare* (OLM) cells of CA1, a class of SST-positive interneurons. Within neuronal nuclei, SLM2 is concentrated in nuclear sub-structures (Figure 1A), reminiscent of nuclear bodies formed by the SLM2 paralog SAM68.[40,41] However, only a fraction of sub-nuclear structures in hippocampal neurons showed SLM2-SAM68 co-localization (Figure 1B). To identify SLM2-associated RNAs, we used enhanced cross-linking and immunoprecipitation (eCLIP) on mouse whole-brain and hippocampal samples. Tag counts obtained from independent replicates with the CTK pipeline[42] were highly correlated (Table S1; Figure S1A). 77% of the binding events occurred in introns, whereas only 2% mapped to exons (Figure 1C). Cross-link-induced truncation site (CITS) analysis identified the exact protein-RNA cross-link sites, which are enriched in the UWAA tetramer element (W = U/A; Figures 1D and S1B), a motif recognized by SLM2 *in vitro*.[43] *De novo* motif discovery using mCross, a computational method to model RBP binding sequence specificity and cross-link sites,[44] revealed a UUWAAAA 7-mer as the dominant RNA motif bound by SLM2 *in vivo* (Figures 1E and S1B–S1D). High–confidence SLM2 binding events in the replicates were identified using CLIPper followed by IDR (Figure 1F; $\log_2$ fold change $\geq$ 2 and false discovery rate [FDR]? $-\log_{10} \geq 2$; Table S1). Gene Ontology analysis of SLM2-bound mRNAs revealed a strong enrichment of mRNAs encoding glutamatergic synapse components (Figures 1G and S1E). Among the 424 high-confidence SLM2 target mRNAs in whole-brain samples, 110 were annotated in SynGO[45] to encode synaptic proteins, with 59 presynaptic and 49 postsynaptic density components (Figure 1H). These include pan-neuronally expressed mRNAs such as *Nrxn1*,*2*,*3*, *Nlgn1*, *Lrrtm4*, *Dlgap1*,*2*, *Tenm2*, and *Cadm1*, as well as postsynaptic proteins preferentially expressed in GABAergic interneurons such as *Erbb4* and *Gria4*.[46,47] No significant peaks were observed in size-matched input samples, and dense clusters of the UWAA motif in target mRNAs often closely aligned with SLM2 binding events (Figure 1I). These experiments uncover an array of mRNAs encoding synaptic proteins that are bound by endogenous SLM2 *in vivo*.

Identification of cell-type-specific SLM2-dependent exons

Action of RBPs is frequently dependent on expression level.[22,48] Thus, we quantified SLM2 immunoreactivity across hippocampal neuron sub-populations (Figures 2A and 2B). SLM2 expression was highest in CA3 pyramidal cells (Grik4-cre; Figure S2A) and significantly expressed in CA1 pyramidal cells (CamK2-cre) and SST interneurons (SST-cre) (Figures 2B and S2B). More than 90% of genetically marked CA1 and CA3 pyramidal cells expressed SLM2 (Figures S2C and S2D). By contrast, SLM2 is not detectable in dentate granule cells (Figure 2B). We performed conditional ablation and mapped transcripts in the respective cell types by RiboTrap.[49,50] Using CamK2-cre, Grik4-cre, and SST-cre lines, we selectively ablated SLM2 in hippocampal CA1 (*Slm2*$^{\Delta CamK2}$) and CA3 (*Slm2*$^{\Delta Grik4}$) pyramidal cells and SST-positive GABAergic interneurons (*Slm2*$^{\Delta SST}$), respectively (Figures 2C and 2D). Immunostaining for SLM2 confirmed complete loss of the protein at postnatal day 16–18 (P16–18) for *Slm2*$^{\Delta SST}$ and P42–45 for *Slm2*$^{\Delta CamK2}$ and *Slm2*$^{\Delta Grik4}$ in 75%–90% of the cre-positive cells (Figure 2D). Using RiboTrap affinity isolation, we deeply mapped the transcriptomes in wild-type and knockout cells (>90 Mio uniquely mapping reads/sample, >84% of reads mapping to mRNA, 4 replicates per genotype and cell population, one replicate for *Slm2*$^{\Delta CamK2}$ excluded due to 3′ bias; see Table S2 for details). There was very little variance between replicates or the RiboTrap samples from knockout versus wild-type mice (Figure 2E), suggesting that loss of SLM2 does not impact the terminal gene batteries of these cell types. Scatterplots further confirmed only minimal alterations at the level of overall gene expression (Figure 2F). The most strongly altered transcript (1.5- to 4.7-fold up-regulated; see Table S2 for details) was the SLM2 paralog SLM1/*Khdrbs2*, consistent with functional cross-repression.[36]

When comparing differential alternative exon usage across wild-type CamK2, Grik4, and SST cells, we identified 2,860 differentially regulated exons between these populations

(H) Sunburst chart and gene examples associated with synaptic function of high-confidence eCLIP targets from whole-brain samples identified by CLIPper and IDR.

(I) SLM2 eCLIP read densities on eCLIP targets compared with size-matched input. UWAA motif enrichment in whole-brain (green) and hippocampal (orange) samples. Coordinates shown are *Grm5* chr7: 87,601,936–87,607,378 and *Cadm1* chr9: 47,836,291–47,840,954.

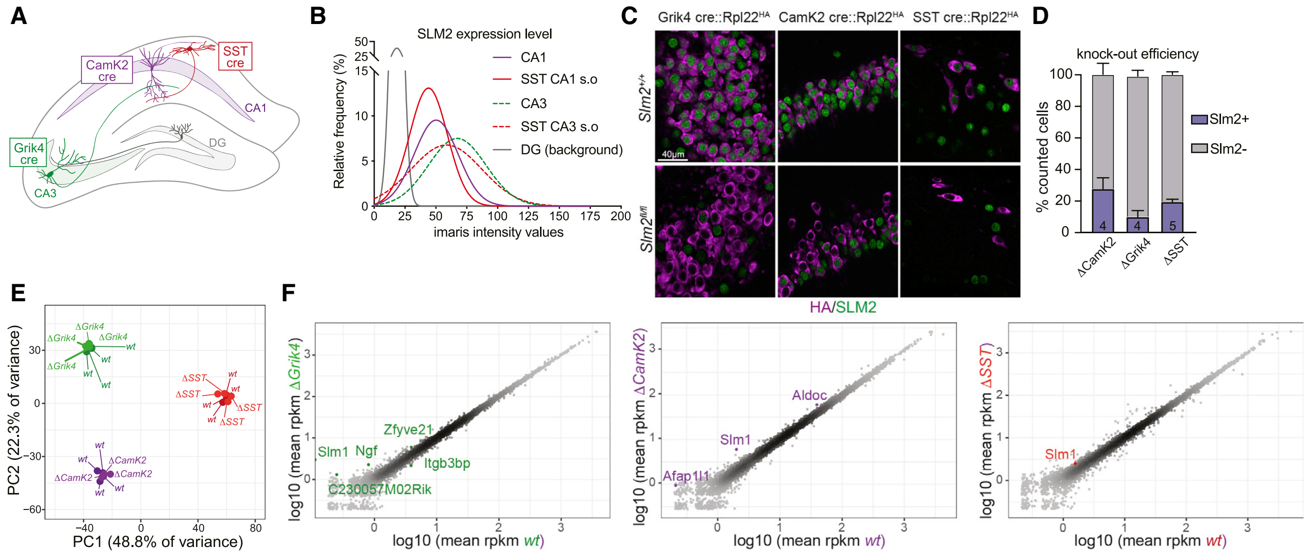

Figure 2. Conditional ablation of SLM2 in hippocampal cell types

(A) Cre drivers to assess the molecular profile of hippocampal *Cornu Ammonis* (CA) 1 (CamK2-cre), CA3 (Grik4-cre), and SST-positive (SST-cre) neurons.

(B) Fitted Gaussian curves of relative frequency of SLM2 immunoreactivity in CA1, CA3 and SST cre-positive neurons in the *stratum oriens* (s.o) of CA1 and CA3. Background immunoreactivity defined based on staining of global $Slm2^{KO}$ mice. n = 3 animals each. CA1: 68 cells, CA3: 75 cells, SST CA1 s.o: 73 cells, SST CA3 s.o: 60 cells, *dentate gyrus* (DG): 62 cells.

(C) SLM2 expression in cre-positive cells defined by immunoreactivity for conditional Rpl22HA allele in $Slm2^{+/+}$ and $Slm2^{fl/fl}$ mice (HA: magenta, SLM2: green, scale bar, 40 μm).

(D) Quantification of SLM2 deletion efficiency at P42–45 in CA1 (ΔCamK2, N = 4, n = 1,081) and CA3 (ΔGrik4, N = 4, n = 1,070) and at P16–18 in SST (ΔSST, N = 5, n = 157) neurons. Mean ± SEM.

(E) Principal-component analysis of genes expressed in hippocampal *Slm2* wild-type and conditional knockout RiboTRAP pull-downs (wild type [WT] in green: $Grik4^{cre}$:$Rpl22^{HA/HA}$ N = 4, Δ*Grik4*: $Grik4^{cre}$::$Rpl22^{HA/HA}$::$Slm2^{fl/fl}$ N = 4; WT in purple: $Camk2^{cre}$::$Rpl22^{HA/HA}$ N = 3, Δ*CamK2*: $Camk2^{cre}$::$Rpl22^{HA/HA}$::$Slm2^{fl/fl}$ N = 3; WT in red: SST^{cre}::$Rpl22^{HA/HA}$ N = 4, Δ*SST*: SST^{cre}::$Rpl22^{HA/HA}$::$Slm2^{fl/fl}$ N = 4). Variances explained by principal component 1 (PC1) and PC2 are indicated. Variance stabilization transformation was utilized to normalize gene expression.

(F) Correlation analysis of the mean log10 transformed, normalized transcript counts (reads per kilobase million [rpkm]) between WT (x axis) and mutants (y axis). Differentially expressed genes (fold change ≥ 1.5, adjusted p value Benjamini and Hochberg ≤ 0.05) are marked in color.

(Table S2). Loss of SLM2 did not broadly modify these cell-type-specific splicing signatures (Figure S3A; Table S2) but modified only a handful of alternative splicing events (Figure 3A; $p \leq 0.01$, fold change ≥ 2; Table S2), including increased exon incorporation at *Nrxn2* alternatively spliced segment 4 in all three cell populations (AS4; see Figures 3A, 3B, and S3E for validation of splicing changes by qPCR and S4A for a sashimi plot). By contrast, the corresponding alternative exon in *Nrxn3* was de-regulated only in CA3 (Grik4) and CA1 (CamK2) cells (Figures 3C and S3E). De-regulation of the mutually exclusive alternative exons e23/e24 in Syntaxin binding protein 5-like (*Stxbp5l*; also called Tomosyn-2) was another splicing event commonly altered in CA1 and CA3 but not $Slm2^{\Delta SST}$ cells (Figures 3A and S3C). In addition, we identified de-regulation of alternative exons in the unconventional myosin 1b (*Myo1b*) (in $Slm2^{\Delta Camk2}$ cells) and the ubiquitin ligase *Ube2d* and the GTPase-activating enzyme *Arhgap42* in $Slm2^{\Delta SST}$ cells.

Integration of the eCLIP and RiboTrap splicing analysis uncovered densely clustered intronic SLM2 binding events and the UWAA motif within 500 bases downstream of the de-regulated alternative exons (Figures 3B and 3C). This demonstrates that SLM2 binding directs skipping of upstream alternative exons. Importantly, no significant eCLIP tags were recovered in *Myo1b*, *Nkd2*, *Ube2d*, and *Arhgap42*, indicating that these mRNAs are not directly regulated by SLM2. Besides these major alterations in a handful of genes, we observed further alterations in alternative exon incorporation in 61 additional mRNAs. The vast majority of these mRNAs are only very lowly expressed (Figure S3B), indicating that the mRNAs are unlikely to have significant contribution to the cellular proteomes. Moreover, no eCLIP binding events were mapped to these mRNAs (Figure S4B). Notably, all directly bound mRNAs with significantly altered alternative splicing encode synaptic proteins.

Loss of SLM2 results in cell-type-specific synaptic phenotypes

Considering the remarkable selectivity of SLM2 for binding and regulating mRNAs encoding synaptic proteins, we probed the functional consequences of its ablation. Global SLM2 knockout is accompanied by increased glutamatergic transmission at CA3-CA1 pyramidal cell Schaffer collateral synapses.[37] Notably, conditional ablation of SLM2 in the presynaptic CA3 pyramidal cells resulted in a significant increase in postsynaptic currents evoked by Schaffer collateral stimulation in CA1 neurons (Figures S5A–S5C). Thus, deletion of SLM2 from CA3 neurons is sufficient to modify synaptic transmission onto postsynaptic CA1 pyramidal cells. We next examined phenotypes resulting from conditional loss of SLM2 in GABAergic interneurons. We

CellPress
OPEN ACCESS

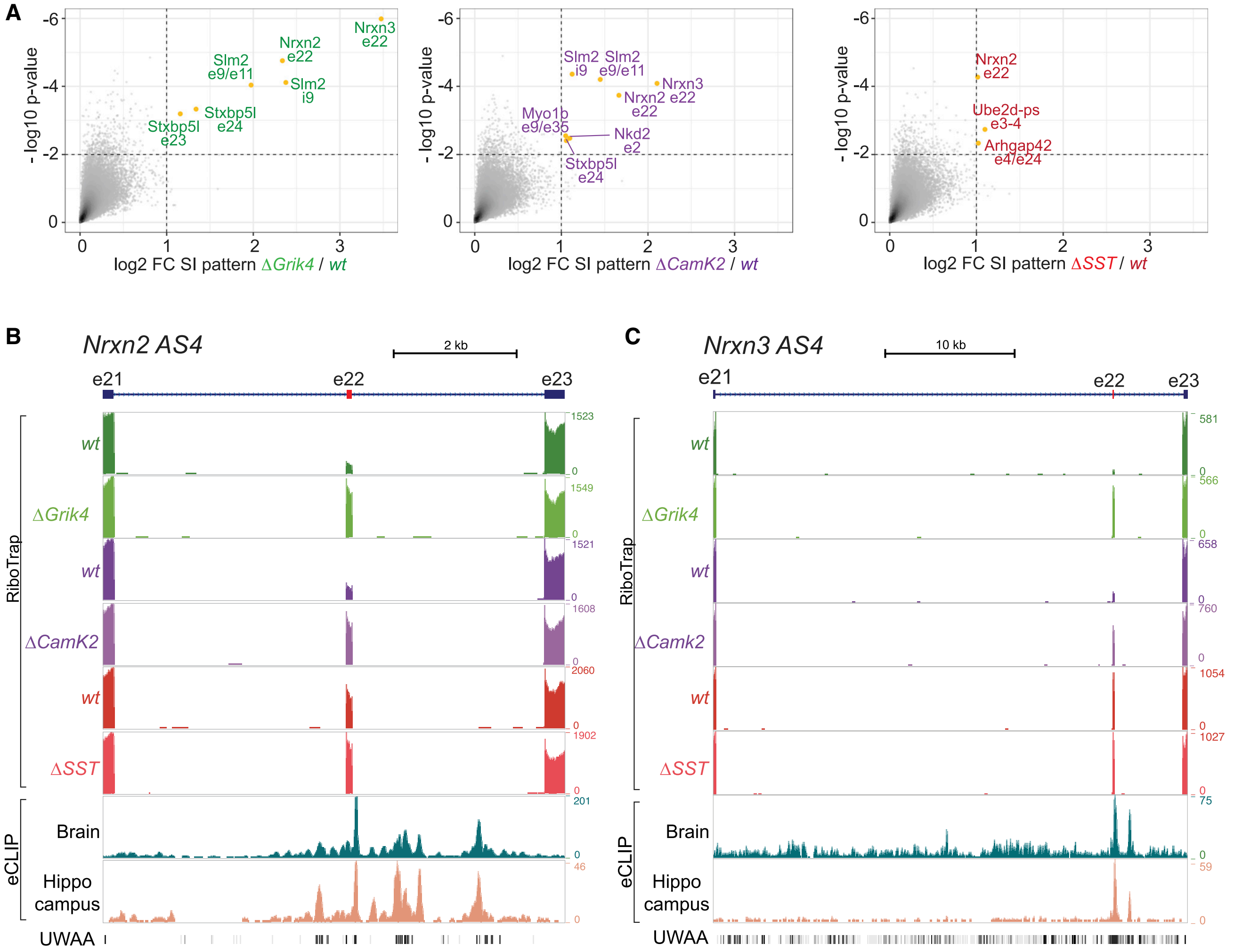

Figure 3. SLM2 directly regulates alternative splicing of mRNAs encoding synaptic proteins

(A) Log2 fold change splicing index (FC SI) and −log10 p values of splicing patterns comparing WT and *Slm2* mutants. Significantly regulated exons are marked in color (fold change ≥ 2, p ≤ 0.01).

(B and C) Sequencing tracks for WT and mutant (Δ) RiboTrap samples for hippocampal Grik4, CamK2, and SST cells and eCLIP analysis in whole brain and hippocampus for de-regulated exons of *Nrxn2* and *Nrxn3*. SLM2 binding events in downstream introns align with UWAA motifs. Coordinates: *Nrxn2* chr19:6,509,778–6,517,248; *Nrxn3* chr12:90,168,920–90,204,818.

focused on horizontally oriented SST interneurons located in the *stratum oriens alveus* of the hippocampus representing putative OLM interneurons, which significantly express SLM2 (Figure S2B). Conditional knockout of SLM2 from SST interneurons ($Slm2^{\Delta SST}$) did not modify intrinsic properties of SST-positive interneurons, indicating that SLM2 is not required for the specification of these cells (Figures S5C–S5F). However, our eCLIP analysis uncovered abundant SLM2 binding to mRNAs that encode proteins of glutamatergic synapses (Figures 1G and 1H). Thus, we examined glutamatergic inputs to $Slm2^{\Delta SST}$ cells. Miniature excitatory postsynaptic current (mEPSC) amplitudes in SST interneurons were unchanged, but we observed a significant shift toward a higher mEPSC frequency, suggesting an increased glutamatergic synapse density onto $Slm2^{\Delta SST}$ cells (Figures 4A–4E). OLM interneuron dendrites in the *stratum oriens* receive glutamatergic synapses from CA1 pyramidal cells.[51] These inputs exhibit a characteristic short-term facilitation, which has a critical impact on hippocampal circuit function.[52,53] We investigated AMPAR-mediated post-synaptic responses with increasing electrical stimulation of putative CA1 axons in the *alveus* and found a significant increase in excitation consistent with a larger synapse number (Figure 4F). Moreover, 40 Hz stimulation of the same axons led to a significantly elevated short-term facilitation (Figure 4G). Because short-term facilitation at this synapse is mediated via increased transmitter release, these results show that SLM2 in postsynaptic SST interneurons controls glutamatergic transmission and synaptic recruitment of these cells via a *trans*-synaptic mechanism.

We further analyzed GABAergic SST interneuron output synapses by optogenetic stimulation of SST interneurons and whole-cell patch-clamp recordings from CA1 pyramidal cells. We found no alterations in the magnitude of optogenetically

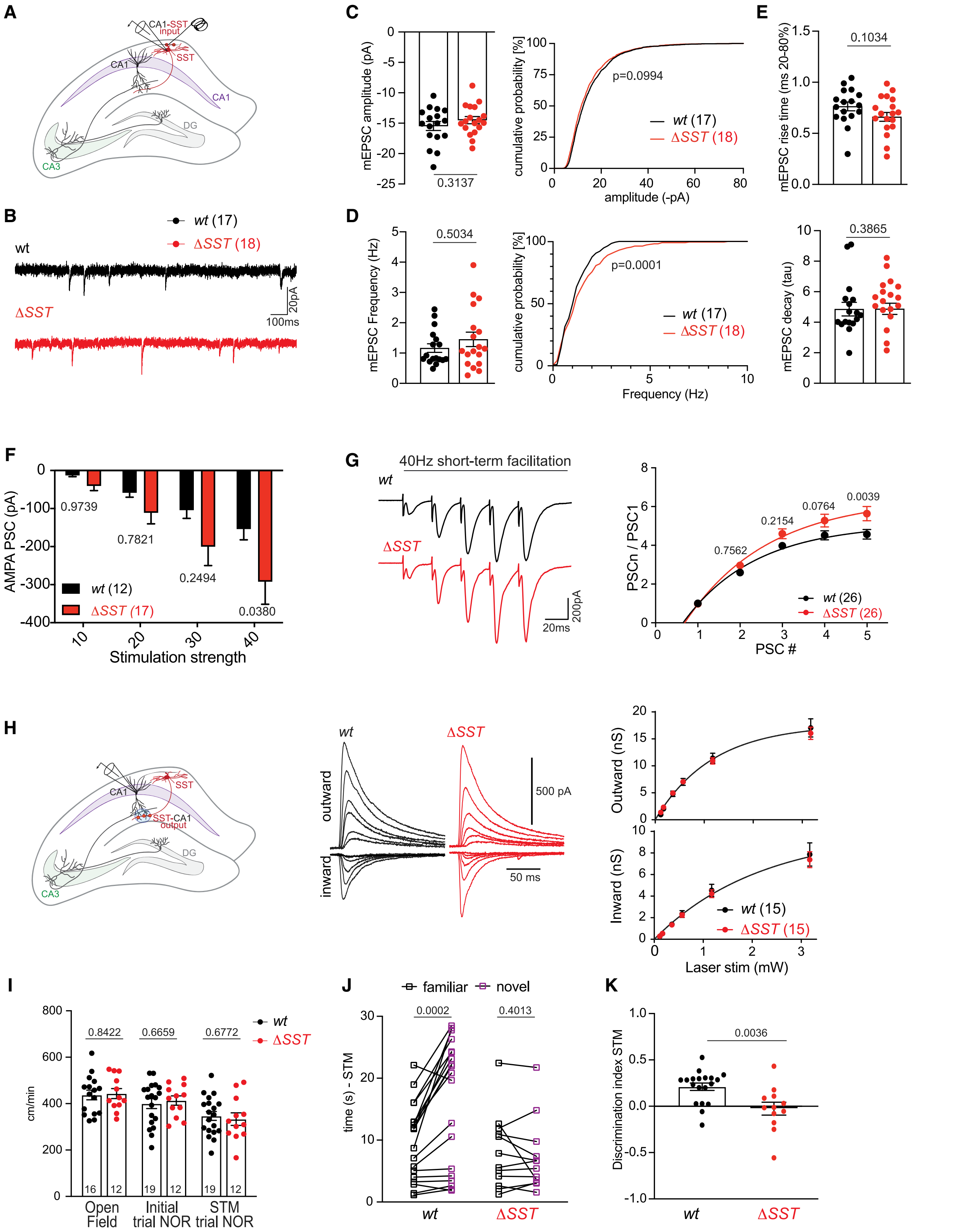

(legend on next page)

evoked postsynaptic inhibitory currents in *Slm2*$^{\Delta SST}$ mice (Figure 4H). The kinetics of optogenetically evoked currents were unchanged, indicating the normal assembly of postsynaptic GABA A receptors (Figure S5G). We applied 10 Hz optogenetic stimulation, which induces a depression at OLM-CA1 synapses in wild-type cells. Using this protocol, we observed a small but significant reduction in short-term depression in slices from *Slm2*$^{\Delta SST}$ mice (Figure S5H). Finally, GABA A receptor kinetics and voltage dependence of GABAergic inhibitory PSCs (IPSCs) were unchanged (Figure S5), suggesting that expression of synaptic GABA A receptor sub-units is virtually identical. Thus, selective loss of SLM2 from SST interneurons results in increased glutamatergic drive onto OLM interneurons with largely similar properties of output synapses onto CA1 neurons.

Aberrant activation of OLM interneurons induced by optogenetic stimulation during the exploration phase has been shown to impair object memory in an object recognition task.[54] Thus, we performed novel object recognition tests with *Slm2*$^{\Delta SST}$ mice. Mutant and wild-type mice did not differ in mobility in the test arena or in the total time spent interacting with objects (Figure 4I). When testing object recognition memory (1 h after the initial object exploration), wild-type mice spent significantly more time exploring the novel object. By contrast, *Slm2*$^{\Delta SST}$ mice spent similar times interacting with novel and familiar objects (Figures 4J and 4K). This defect in short-term memory was not associated with an increase in anxiety, as *Slm2*$^{\Delta SST}$ mice showed normal exploration of open and closed arms in elevated plus maze and also did not differ in other behavioral assessments such as marble burying (Figure S6). Thus, selective loss of SLM2 from SST interneurons is associated with a specific deficit in short-term memory in mice. Taken together, these data suggest that in SST interneurons, SLM2 controls splicing of a very small sub-set of mRNAs encoding synaptic proteins including *Nrxn2*, leading to the regulation of the glutamatergic recruitment of these GABAergic interneurons for fine-tuning of dendritic inhibition during learning and memory.

DISCUSSION

In the nervous system, alternative splicing controls multiple steps of neuronal development, plasticity, and diverse pathologies.[12,14,16,19,55–57] Here, we discovered that SLM2 is dispensable for most aspects of neuronal differentiation but selectively instructs terminal specification of synaptic function within the hippocampal microcircuit.

We demonstrate that endogenous SLM2 binds to a UUWAAAA 7-mer motif *in vivo*. The SLM2 paralog SAM68 recognizes a similar motif; however, direct comparison of alternative splicing profiles in *Slm2*^{KO} and *Sam68*^{KO} hippocampi suggests that de-regulated exons are largely paralog specific *in vivo* (Figure S4C). SLM2 eCLIP targets are strongly enriched for mRNAs encoding synaptic proteins, including adhesion molecules, pre- and postsynaptic scaffolding molecules, and neurotransmitter receptors. Interestingly, only a small fraction of these SLM2-bound mRNAs exhibit alterations in alternative exon incorporation in conditional knockout mice. This might be a consequence of functional redundancy with other RBPs. Alternatively, SLM2 binding to target mRNAs in the nucleus may contribute to coordinated spatiotemporal control of an array of functionally related mRNAs, which modifies their trafficking and/or translation.[58] For all regulated alternative exons, SLM2 binding sites consist of extended RNA motif clusters in the downstream intron, and loss of SLM2 results in aberrant exon incorporation. This indicates a major function for SLM2 in driving exon skipping.

Interestingly, loss of SLM2 did not result in significant alterations in the overall neuronal transcriptomes or functional intrinsic properties. This strongly suggests that SLM2 is dispensable for cell fate specification. By contrast, SLM2 loss of function was associated with selective *trans*-synaptic phenotypes: Conditional ablation of SLM2 from CA3 pyramidal neurons led to an increase in postsynaptic currents at Schaffer collateral synapses onto CA1. This phenotype recapitulates the increase in postsynaptic AMPA receptors and increased synaptic transmission observed in global *Slm2*^{KO} mice.[37] In SST interneurons, conditional *Slm2* deletion was associated with increased glutamatergic transmission likely resulting from an increased glutamatergic synapse density onto the mutant cells and increased presynaptic facilitation of synapses formed onto the knockout cells. SLM2-dependent alterations in synaptic adhesion molecules in SST interneurons, such as TENM2, ERBB4, CADM1, ADGRL3, and postsynaptic NRXNs are well positioned to direct such *trans*-synaptic regulation.[46,59,60] For example, the elevated alternative exon incorporation in NRXN2 AS4 is predicted to reduce its ability to inactivate the function of postsynaptic neuroligins

Figure 4. SLM2 controls synaptic plasticity and function in a *trans*-synaptic manner

(A) Electrical stimulation of axons in the *alveus* and voltage-clamp recordings in genetically marked SST-positive interneurons in the s.o.

(B) Example traces of miniature excitatory postsynaptic currents (mEPSCs) in SST interneurons.

(C–E) Analysis of mEPSC amplitude (C), frequency (D), and rise and decay times (E). WT n = 17, Δ*SST* n = 18. Mean ± SEM. p values determined by corresponding t tests. For cumulative frequency distribution, Kolmogorov-Smirnov test.

(F) Mean ± SEM data of AMPAR-mediated EPSCs in SST interneurons of s.o in response to different stimulation intensities of axons in the alveus in Δ*SST* n = 17 versus WT n = 12. Two-way ANOVA with Šídák's multiple comparisons test.

(G) Representative traces of evoked EPSCs during repetitive stimulation at 40 Hz in WT (black) and Δ*SST* (red). Group data of EPSCs normalized to the first peak in Δ*SST* n = 26 versus WT n = 26. Mean ± SEM. Two-way ANOVA with Šídák's multiple comparisons test.

(H) Local optogenetic stimulation of SST+ neuron-mediated IPSCs in apical CA1 dendrites in WT and Δ*SST* mutants. Representative traces of inward and outward IPSCs evoked at increasing laser intensities in WT (black) and Δ*SST* (red). Mean ± SEM input-output curve of synaptic conductance underlying outward and inward PSCs.

(I) Quantification of velocity (cm/min) in open field, initial, and short-term memory (STM) test phases of novel object recognition (NOR) task. Animal numbers for each task are indicated. Mean ± SEM, unpaired t test.

(J and K) NOR interaction time (s) spent with a familiar (black) or novel (purple) object during a 5 min trial (paired t test) and discrimination index (unpaired t test). Mean ± SEM, WT n = 19 and Δ*SST* n = 12.

in neuronal dendrites[61,62] and may contribute to the increased glutamatergic input received by OLM interneurons in *Slm2*$^{\Delta SST}$ mice. Thus, conditional SLM2 ablation reconfigures *trans*-synaptic interaction modules and, thereby, properties of synaptic structures formed with connecting cells.

Despite the increased glutamatergic drive received by OLM interneurons, their GABAergic output was largely unchanged in *Slm2*$^{\Delta SST}$ mice. This suggests that SLM2 regulates the level of functional recruitment of SST interneurons. SST interneurons provide branch-specific inhibition onto distal dendrites of CA1 pyramidal cells, powerfully controlling dendritic integration of synaptic information.[63] Increased activation of OLM interneurons during the formation of episodic memories has been shown to disrupt memory formation.[54] Consistent with aberrant activation of OLM interneurons, SLM2 knockout mice exhibit an impairment in short-term memory, supporting a critical function for SLM2 in the inhibitory control of short-term episodic memories.

We propose that acquisition of a cell-type-specific complement of RBPs represents a critical element of the terminal gene battery established during development that shapes *trans*-synaptic modules.[4] Expression of SLM2 in a sub-class of SST interneurons[64] and in hippocampal pyramidal cells is already detected at embryonic stages (Figure S2F). Thus, SLM2 expression is linked to embryonic cell-type specification. Evolutionary comparisons of synaptic building blocks across organisms suggest that more complex cellular modules accommodate the need for phenotypic diversity at the level of individual synapses.[65] Our work suggests that the modification of synaptic modules through alternative splicing is a major mechanism underlying the unique functional specification of synaptic connections.

Limitations of the study

While our study correlates alterations in alternative splicing and synaptic transmission phenotypes, we have not directly linked a single alternative splice isoform of a synaptic protein to the alteration in plasticity. Moreover, we cannot exclude a contribution of SST interneuron populations besides OLM cells to the behavioral phenotype.

STAR★METHODS

Detailed methods are provided in the online version of this paper and include the following:

- KEY RESOURCES TABLE
- RESOURCE AVAILABILITY
  - ○ Lead contact
  - ○ Materials availability
  - ○ Data and code availability
- EXPERIMENTAL MODEL AND SUBJECT DETAILS
- METHOD DETAILS
  - ○ Mice
  - ○ Antibodies
  - ○ Primary hippocampal cell culture
  - ○ Immunohistochemistry, image acquisition and statistical analysis
  - ○ eCLIP library preparation
  - ○ eCLIP data processing
  - ○ Motif analysis
  - ○ Gene ontology analysis
  - ○ RiboTRAP pulldowns, RNA purification and quality control
  - ○ Library preparation and illumina sequencing
  - ○ Quality control and RNA-seq pre-processing
  - ○ Differential gene expression analysis
  - ○ Alternative splicing analysis
  - ○ qPCR analysis for alternative exon usage of *Nrxns* at AS4
  - ○ Electrophysiology
  - ○ Behavioral analysis
- QUANTIFICATION AND STATISTICAL ANALYSIS

SUPPLEMENTAL INFORMATION

Supplemental information can be found online at https://doi.org/10.1016/j.celrep.2023.112173.

ACKNOWLEDGMENTS

We thank Caroline Bornmann and Sabrina Innocenti for experimental support and Geoffrey Fucile (SciCORE), the Biozentrum Imaging Core Facility, and Quantitative Genomics Center Basel for excellent support. This work was supported by funds to P.S. from University of Basel, the Swiss National Science Foundation (project 179432, TMAG-3-209273), ERC Advanced Grant SPLICECODE, and AIMS-2-TRIALS, which are supported by the Innovative Medicines Initiatives from the European Commission, funds to J.B. (SNSF, project 176321) and C.Z. (National Institutes of Health, R01NS125018).

AUTHOR CONTRIBUTIONS

L.T., J.S., A.M.G., and D.S. conducted experiments and analyzed data; R.O., H.F., and E.F. analyzed data; L.T., J.S., A.M.G., D.S., J.B., C.Z., and P.S. designed experiments; L.T. and P.S. wrote the manuscript with editing provided by all co-authors.

DECLARATION OF INTERESTS

The authors declare no competing interests.

INCLUSION AND DIVERSITY

We support inclusive, diverse, and equitable conduct of research.

Received: July 22, 2022
Revised: December 7, 2022
Accepted: February 12, 2023
Published: March 1, 2023

REFERENCES

1. Reyes, A., Lujan, R., Rozov, A., Burnashev, N., Somogyi, P., and Sakmann, B. (1998). Target-cell-specific facilitation and depression in neocortical circuits. Nat. Neurosci. *1*, 279–285. https://doi.org/10.1038/1092.
2. Koester, H.J., and Johnston, D. (2005). Target cell-dependent normalization of transmitter release at neocortical synapses. Science *308*, 863–866. https://doi.org/10.1126/science.1100815.
3. Éltes, T., Kirizs, T., Nusser, Z., and Holderith, N. (2017). Target cell type-dependent differences in Ca(2+) channel function underlie distinct release probabilities at hippocampal glutamatergic terminals. J. Neurosci. *37*, 1910–1924. https://doi.org/10.1523/JNEUROSCI.2024-16.2017.

CellPress
OPEN ACCESS

4. Gomez, A.M., Traunmüller, L., and Scheiffele, P. (2021). Neurexins: molecular codes for shaping neuronal synapses. Nat. Rev. Neurosci. *22*, 137–151. https://doi.org/10.1038/s41583-020-00415-7.

5. Apóstolo, N., and de Wit, J. (2019). Compartmentalized distributions of neuronal and glial cell-surface proteins pattern the synaptic network. Curr. Opin. Neurobiol. *57*, 126–133. https://doi.org/10.1016/j.conb.2019.01.025.

6. Südhof, T.C. (2017). Synaptic neurexin complexes: a molecular code for the logic of neural circuits. Cell *171*, 745–769. https://doi.org/10.1016/j.cell.2017.10.024.

7. Yuzaki, M. (2018). Two classes of secreted synaptic organizers in the central nervous system. Annu. Rev. Physiol. *80*, 243–262. https://doi.org/10.1146/annurev-physiol-021317-121322.

8. Krueger-Burg, D., Papadopoulos, T., and Brose, N. (2017). Organizers of inhibitory synapses come of age. Curr. Opin. Neurobiol. *45*, 66–77. https://doi.org/10.1016/j.conb.2017.04.003.

9. Chowdhury, D., Watters, K., and Biederer, T. (2021). Synaptic recognition molecules in development and disease. Curr. Top. Dev. Biol. *142*, 319–370. https://doi.org/10.1016/bs.ctdb.2020.12.009.

10. Favuzzi, E., and Rico, B. (2018). Molecular diversity underlying cortical excitatory and inhibitory synapse development. Curr. Opin. Neurobiol. *53*, 8–15. https://doi.org/10.1016/j.conb.2018.03.011.

11. Siddiqui, T.J., and Craig, A.M. (2011). Synaptic organizing complexes. Curr. Opin. Neurobiol. *21*, 132–143. https://doi.org/10.1016/j.conb.2010.08.016.

12. Furlanis, E., and Scheiffele, P. (2018). Regulation of neuronal differentiation, function, and plasticity by alternative splicing. Annu. Rev. Cell Dev. Biol. *34*, 451–469.

13. Ule, J., and Blencowe, B.J. (2019). Alternative splicing regulatory networks: functions, mechanisms, and evolution. Mol. Cell *76*, 329–345. https://doi.org/10.1016/j.molcel.2019.09.017.

14. Vuong, C.K., Black, D.L., and Zheng, S. (2016). The neurogenetics of alternative splicing. Nat. Rev. Neurosci. *17*, 265–281. https://doi.org/10.1038/nrn.2016.27.

15. Merkin, J., Russell, C., Chen, P., and Burge, C.B. (2012). Evolutionary dynamics of gene and isoform regulation in Mammalian tissues. Science *338*, 1593–1599. https://doi.org/10.1126/science.1228186.

16. Barbosa-Morais, N.L., Irimia, M., Pan, Q., Xiong, H.Y., Gueroussov, S., Lee, L.J., Slobodeniuc, V., Kutter, C., Watt, S., Colak, R., and Blencowe, B.J. (2012). The evolutionary landscape of alternative splicing in vertebrate species. Science *338*, 1587–1593. https://doi.org/10.1111/aman.12147.

17. Mazin, P.V., Khaitovich, P., Cardoso-Moreira, M., and Kaessmann, H. (2021). Alternative splicing during mammalian organ development. Nat. Genet. *53*, 925–934. https://doi.org/10.1038/s41588-021-00851-w.

18. Grosso, A.R., Gomes, A.Q., Barbosa-Morais, N.L., Caldeira, S., Thorne, N.P., Grech, G., von Lindern, M., and Carmo-Fonseca, M. (2008). Tissue-specific splicing factor gene expression signatures. Nucleic Acids Res. *36*, 4823–4832. https://doi.org/10.1093/nar/gkn463.

19. Zhang, X., Chen, M.H., Wu, X., Kodani, A., Fan, J., Doan, R., Ozawa, M., Ma, J., Yoshida, N., Reiter, J.F., et al. (2016). Cell-type-specific alternative splicing governs cell fate in the developing cerebral cortex. Cell *166*, 1147–1162.e15. https://doi.org/10.1016/j.cell.2016.07.025.

20. Weyn-Vanhentenryck, S.M., Feng, H., Ustianenko, D., Duffié, R., Yan, Q., Jacko, M., Martinez, J.C., Goodwin, M., Zhang, X., Hengst, U., et al. (2018). Precise temporal regulation of alternative splicing during neural development. Nat. Commun. *9*, 2189. https://doi.org/10.1038/s41467-018-04559-0.

21. Furlanis, E., Traunmüller, L., Fucile, G., and Scheiffele, P. (2019). Landscape of ribosome-engaged transcript isoforms reveals extensive neuronal-cell-class-specific alternative splicing programs. Nat. Neurosci. *22*, 1709–1717. https://doi.org/10.1038/s41593-019-0465-5.

22. Feng, H., Moakley, D.F., Chen, S., McKenzie, M.G., Menon, V., and Zhang, C. (2021). Complexity and graded regulation of neuronal cell-type-specific alternative splicing revealed by single-cell RNA sequencing. Proc. Natl. Acad. Sci. USA *118*, e2013056118. https://doi.org/10.1073/pnas.2013056118.

23. Ray, T.A., Cochran, K., Kozlowski, C., Wang, J., Alexander, G., Cady, M.A., Spencer, W.J., Ruzycki, P.A., Clark, B.S., Laeremans, A., et al. (2020). Comprehensive identification of mRNA isoforms reveals the diversity of neural cell-surface molecules with roles in retinal development and disease. Nat. Commun. *11*, 3328. https://doi.org/10.1038/s41467-020-17009-7.

24. Hauser, D., Behr, K., Konno, K., Schreiner, D., Schmidt, A., Watanabe, M., Bischofberger, J., and Scheiffele, P. (2022). Targeted proteoform mapping uncovers specific Neurexin-3 variants required for dendritic inhibition. Neuron *110*, 2094–2109.e10. https://doi.org/10.1016/j.neuron.2022.04.017.

25. Wamsley, B., Jaglin, X.H., Favuzzi, E., Quattrocolo, G., Nigro, M.J., Yusuf, N., Khodadadi-Jamayran, A., Rudy, B., and Fishell, G. (2018). Rbfox1 mediates cell-type-specific splicing in cortical interneurons. Neuron *100*, 846–859.e7. https://doi.org/10.1016/j.neuron.2018.09.026.

26. Irimia, M., Weatheritt, R.J., Ellis, J.D., Parikshak, N.N., Gonatopoulos-Pournatzis, T., Babor, M., Quesnel-Vallières, M., Tapial, J., Raj, B., O'Hanlon, D., et al. (2014). A highly conserved program of neuronal microexons is misregulated in autistic brains. Cell *159*, 1511–1523. https://doi.org/10.1016/j.cell.2014.11.035.

27. Quesnel-Vallières, M., Dargaei, Z., Irimia, M., Gonatopoulos-Pournatzis, T., Ip, J.Y., Wu, M., Sterne-Weiler, T., Nakagawa, S., Woodin, M.A., Blencowe, B.J., and Cordes, S.P. (2016). Misregulation of an activity-dependent splicing network as a common mechanism underlying autism spectrum disorders. Mol. Cell *64*, 1023–1034. https://doi.org/10.1016/j.molcel.2016.11.033.

28. Parikshak, N.N., Swarup, V., Belgard, T.G., Irimia, M., Ramaswami, G., Gandal, M.J., Hartl, C., Leppa, V., Ubieta, L.d.l.T., Huang, J., et al. (2016). Genome-wide changes in lncRNA, splicing, and regional gene expression patterns in autism. Nature *540*, 423–427. https://doi.org/10.1038/nature20612.

29. Torres-Méndez, A., Pop, S., Bonnal, S., Almudi, I., Avola, A., Roberts, R.J.V., Paolantoni, C., Alcaina-Caro, A., Martín-Anduaga, A., Haussmann, I.U., et al. (2022). Parallel evolution of a splicing program controlling neuronal excitability in flies and mammals. Sci. Adv. *8*, eabk0445. https://doi.org/10.1126/sciadv.abk0445.

30. Li, Q., Zheng, S., Han, A., Lin, C.H., Stoilov, P., Fu, X.D., and Black, D.L. (2014). The splicing regulator PTBP2 controls a program of embryonic splicing required for neuronal maturation. Elife *3*, e01201. https://doi.org/10.7554/eLife.01201.

31. Saito, Y., Yuan, Y., Zucker-Scharff, I., Fak, J.J., Jereb, S., Tajima, Y., Licatalosi, D.D., and Darnell, R.B. (2019). Differential NOVA2-mediated splicing in excitatory and inhibitory neurons regulates cortical development and cerebellar function. Neuron *101*, 707–720.e5. https://doi.org/10.1016/j.neuron.2018.12.019.

32. Vuong, C.K., Wei, W., Lee, J.A., Lin, C.H., Damianov, A., de la Torre-Ubieta, L., Halabi, R., Otis, K.O., Martin, K.C., O'Dell, T.J., and Black, D.L. (2018). Rbfox1 regulates synaptic transmission through the inhibitory neuron-specific vSNARE Vamp1. Neuron *98*, 127–141.e7. https://doi.org/10.1016/j.neuron.2018.03.008.

33. Jacko, M., Weyn-Vanhentenryck, S.M., Smerdon, J.W., Yan, R., Feng, H., Williams, D.J., Pai, J., Xu, K., Wichterle, H., and Zhang, C. (2018). Rbfox splicing factors promote neuronal maturation and axon initial segment assembly. Neuron *97*, 853–868.e6. https://doi.org/10.1016/j.neuron.2018.01.020.

34. Stoss, O., Novoyatleva, T., Gencheva, M., Olbrich, M., Benderska, N., and Stamm, S. (2004). p59(fyn)-mediated phosphorylation regulates the activity of the tissue-specific splicing factor rSLM-1. Mol. Cell. Neurosci. *27*, 8–21. https://doi.org/10.1016/j.mcn.2004.04.011 S1044743104001010.

35. Iijima, T., Iijima, Y., Witte, H., and Scheiffele, P. (2014). Neuronal cell type-specific alternative splicing is regulated by the KH domain protein SLM1. J. Cell Biol. *204*, 331–342. https://doi.org/10.1083/jcb.201310136.

36. Traunmüller, L., Bornmann, C., and Scheiffele, P. (2014). Alternative splicing coupled nonsense-mediated decay generates neuronal cell type-specific expression of SLM proteins. J. Neurosci. *34*, 16755–16761. https://doi.org/10.1523/JNEUROSCI.3395-14.2014.

37. Traunmüller, L., Gomez, A.M., Nguyen, T.-M., and Scheiffele, P. (2016). Control of neuronal synapse specification by highly dedicated alternative splicing program. Science *352*, 982–986.

38. Ehrmann, I., Gazzara, M.R., Pagliarini, V., Dalgliesh, C., Kheirollahi-Chadegani, M., Xu, Y., Cesari, E., Danilenko, M., Maclennan, M., Lowdon, K., et al. (2016). A SLM2 feedback pathway controls cortical network activity and mouse behavior. Cell Rep. *17*, 3269–3280. https://doi.org/10.1016/j.celrep.2016.12.002.

39. Nguyen, T.M., Schreiner, D., Xiao, L., Traunmüller, L., Bornmann, C., and Scheiffele, P. (2016). An alternative splicing switch shapes neurexin repertoires in principal neurons versus interneurons in the mouse hippocampus. Elife *5*, e22757. https://doi.org/10.7554/eLife.22757.

40. Chen, T., Boisvert, F.M., Bazett-Jones, D.P., and Richard, S. (1999). A role for the GSG domain in localizing Sam68 to novel nuclear structures in cancer cell lines. Mol. Biol. Cell *10*, 3015–3033. https://doi.org/10.1091/mbc.10.9.3015.

41. Mannen, T., Yamashita, S., Tomita, K., Goshima, N., and Hirose, T. (2016). The Sam68 nuclear body is composed of two RNase-sensitive substructures joined by the adaptor HNRNPL. J. Cell Biol. *214*, 45–59. https://doi.org/10.1083/jcb.201601024.

42. Shah, A., Qian, Y., Weyn-Vanhentenryck, S.M., and Zhang, C. (2017). CLIP Tool Kit (CTK): a flexible and robust pipeline to analyze CLIP sequencing data. Bioinformatics *33*, 566–567. https://doi.org/10.1093/bioinformatics/btw653.

43. Danilenko, M., Dalgliesh, C., Pagliarini, V., Naro, C., Ehrmann, I., Feracci, M., Kheirollahi-Chadegani, M., Tyson-Capper, A., Clowry, G.J., Fort, P., et al. (2017). Binding site density enables paralog-specific activity of SLM2 and Sam68 proteins in Neurexin2 AS4 splicing control. Nucleic Acids Res. *45*, 4120–4130. https://doi.org/10.1093/nar/gkw1277.

44. Feng, H., Bao, S., Rahman, M.A., Weyn-Vanhentenryck, S.M., Khan, A., Wong, J., Shah, A., Flynn, E.D., Krainer, A.R., and Zhang, C. (2019). Modeling RNA-binding protein specificity in vivo by precisely registering protein-RNA crosslink sites. Mol. Cell *74*, 1189–1204.e6. https://doi.org/10.1016/j.molcel.2019.02.002.

45. Koopmans, F., van Nierop, P., Andres-Alonso, M., Byrnes, A., Cijsouw, T., Coba, M.P., Cornelisse, L.N., Farrell, R.J., Goldschmidt, H.L., Howrigan, D.P., et al. (2019). SynGO: an evidence-based, expert-curated knowledge base for the synapse. Neuron *103*, 217–234.e4. https://doi.org/10.1016/j.neuron.2019.05.002.

46. Fazzari, P., Paternain, A.V., Valiente, M., Pla, R., Luján, R., Lloyd, K., Lerma, J., Marín, O., and Rico, B. (2010). Control of cortical GABA circuitry development by Nrg1 and ErbB4 signalling. Nature *464*, 1376–1380. https://doi.org/10.1038/nature08928.

47. Geiger, J.R., Melcher, T., Koh, D.S., Sakmann, B., Seeburg, P.H., Jonas, P., and Monyer, H. (1995). Relative abundance of subunit mRNAs determines gating and Ca2+ permeability of AMPA receptors in principal neurons and interneurons in rat CNS. Neuron *15*, 193–204. https://doi.org/10.1016/0896-6273(95)90076-4.

48. Begg, B.E., Jens, M., Wang, P.Y., Minor, C.M., and Burge, C.B. (2020). Concentration-dependent splicing is enabled by Rbfox motifs of intermediate affinity. Nat. Struct. Mol. Biol. *27*, 901–912. https://doi.org/10.1038/s41594-020-0475-8.

49. Sanz, E., Yang, L., Su, T., Morris, D.R., McKnight, G.S., and Amieux, P.S. (2009). Cell-type-specific isolation of ribosome-associated mRNA from complex tissues. Proc. Natl. Acad. Sci. USA *106*, 13939–13944. https://doi.org/10.1073/pnas.0907143106.

50. Heiman, M., Kulicke, R., Fenster, R.J., Greengard, P., and Heintz, N. (2014). Cell type-specific mRNA purification by translating ribosome affinity purification (TRAP). Nat. Protoc. *9*, 1282–1291. https://doi.org/10.1038/nprot.2014.085.

51. Somogyi, P., and Klausberger, T. (2005). Defined types of cortical interneurone structure space and spike timing in the hippocampus. J. Physiol. *562*, 9–26. https://doi.org/10.1113/jphysiol.2004.078915.

52. Ali, A.B., and Thomson, A.M. (1998). Facilitating pyramid to horizontal oriens-alveus interneurone inputs: dual intracellular recordings in slices of rat hippocampus. J. Physiol. *507*, 185–199. https://doi.org/10.1111/j.1469-7793.1998.185bu.x.

53. Pouille, F., and Scanziani, M. (2004). Routing of spike series by dynamic circuits in the hippocampus. Nature *429*, 717–723. https://doi.org/10.1038/nature02615.

54. Siwani, S., França, A.S.C., Mikulovic, S., Reis, A., Hilscher, M.M., Edwards, S.J., Leão, R.N., Tort, A.B.L., and Kullander, K. (2018). OLMalpha2 cells bidirectionally modulate learning. Neuron *99*, 404–412.e3. https://doi.org/10.1016/j.neuron.2018.06.022.

55. Raj, B., and Blencowe, B.J. (2015). Alternative splicing in the mammalian nervous system: recent insights into mechanisms and functional roles. Neuron *87*, 14–27. https://doi.org/10.1016/j.neuron.2015.05.004.

56. Jensen, K.B., Dredge, B.K., Stefani, G., Zhong, R., Buckanovich, R.J., Okano, H.J., Yang, Y.Y., and Darnell, R.B. (2000). Nova-1 regulates neuron-specific alternative splicing and is essential for neuronal viability. Neuron *25*, 359–371. https://doi.org/10.1016/s0896-6273(00)80900-9.

57. Johnson, V., Junge, H.J., and Chen, Z. (2019). Temporal regulation of axonal repulsion by alternative splicing of a conserved microexon in mammalian Robo1 and Robo2. Elife *8*, e46042. https://doi.org/10.7554/eLife.46042.

58. Chen, X., and Mayr, C. (2022). A working model for condensate RNA-binding proteins as matchmakers for protein complex assembly. RNA *28*, 76–87. https://doi.org/10.1261/rna.078995.121.

59. Park, K.A., Ribic, A., Laage Gaupp, F.M., Coman, D., Huang, Y., Dulla, C.G., Hyder, F., and Biederer, T. (2016). Excitatory synaptic drive and feedforward inhibition in the hippocampal CA3 circuit are regulated by SynCAM 1. J. Neurosci. *36*, 7464–7475. https://doi.org/10.1523/JNEUROSCI.0189-16.2016.

60. Ribeiro, L.F., Verpoort, B., Nys, J., Vennekens, K.M., Wierda, K.D., and de Wit, J. (2019). SorCS1-mediated sorting in dendrites maintains neurexin axonal surface polarization required for synaptic function. PLoS Biol. *17*, e3000466. https://doi.org/10.1371/journal.pbio.3000466.

61. Taniguchi, H., Gollan, L., Scholl, F.G., Mahadomrongkul, V., Dobler, E., Limthong, N., Peck, M., Aoki, C., and Scheiffele, P. (2007). Silencing of neuroligin function by postsynaptic neurexins. J. Neurosci. *27*, 2815–2824.

62. Wang, C.Y., Trotter, J.H., Liakath-Ali, K., Lee, S.J., Liu, X., and Südhof, T.C. (2021). Molecular self-avoidance in synaptic neurexin complexes. Sci. Adv. *7*, eabk1924. https://doi.org/10.1126/sciadv.abk1924.

63. Schulz, J.M., Knoflach, F., Hernandez, M.C., and Bischofberger, J. (2018). Dendrite-targeting interneurons control synaptic NMDA-receptor activation via nonlinear alpha5-GABAA receptors. Nat. Commun. *9*, 3576. https://doi.org/10.1038/s41467-018-06004-8.

64. Lim, L., Pakan, J.M.P., Selten, M.M., Marques-Smith, A., Llorca, A., Bae, S.E., Rochefort, N.L., and Marín, O. (2018). Optimization of interneuron function by direct coupling of cell migration and axonal targeting. Nat. Neurosci. *21*, 920–931. https://doi.org/10.1038/s41593-018-0162-9.

65. Arendt, D. (2020). The evolutionary assembly of neuronal machinery. Curr. Biol. *30*, R603–R616. https://doi.org/10.1016/j.cub.2020.04.008.

66. Witte, H., Schreiner, D., and Scheiffele, P. (2019). A Sam68-dependent alternative splicing program shapes postsynaptic protein complexes. Eur. J. Neurosci. *49*, 1436–1453. https://doi.org/10.1111/ejn.14332.

67. Schindelin, J., Arganda-Carreras, I., Frise, E., Kaynig, V., Longair, M., Pietzsch, T., Preibisch, S., Rueden, C., Saalfeld, S., Schmid, B., et al.

CellPress
OPEN ACCESS

(2012). Fiji: an open-source platform for biological-image analysis. Nat. Methods *9*, 676–682. https://doi.org/10.1038/nmeth.2019.

68. Liao, Y., Smyth, G.K., and Shi, W. (2014). featureCounts: an efficient general purpose program for assigning sequence reads to genomic features. Bioinformatics *30*, 923–930. https://doi.org/10.1093/bioinformatics/btt656.
69. Li, H., Handsaker, B., Wysoker, A., Fennell, T., Ruan, J., Homer, N., Marth, G., Abecasis, G., and Durbin, R.; 1000 Genome Project Data Processing Subgroup (2009). The sequence alignment/map format and SAMtools. Bioinformatics *25*, 2078–2079. https://doi.org/10.1093/bioinformatics/btp352.
70. Dobin, A., Davis, C.A., Schlesinger, F., Drenkow, J., Zaleski, C., Jha, S., Batut, P., Chaisson, M., and Gingeras, T.R. (2013). STAR: ultrafast universal RNA-seq aligner. Bioinformatics *29*, 15–21. https://doi.org/10.1093/bioinformatics/bts635.
71. Love, M.I., Huber, W., and Anders, S. (2014). Moderated estimation of fold change and dispersion for RNA-seq data with DESeq2. Genome Biol. *15*, 550. https://doi.org/10.1186/s13059-014-0550-8.
72. Yeo, G.W., Coufal, N.G., Liang, T.Y., Peng, G.E., Fu, X.D., and Gage, F.H. (2009). An RNA code for the FOX2 splicing regulator revealed by mapping RNA-protein interactions in stem cells. Nat. Struct. Mol. Biol. *16*, 130–137. https://doi.org/10.1038/nsmb.1545.
73. Li, Q., Brown, J.B., Huang, H., and Bickel, P.J. (2011). Measuring reproducibility of high-throughput experiments. Ann. Appl. Stat. *5*, 1752–1779.
74. Zhang, Y., Zhou, R., and Wang, Y. (2022). Sashimi.py: a flexible toolkit for combinatorial analysis of genomic data. Preprint at bioRxiv. https://doi.org/10.1101/2022.11.02.514803.
75. Huang, D.W., Sherman, B.T., and Lempicki, R.A. (2009). Systematic and integrative analysis of large gene lists using DAVID bioinformatics resources. Nat. Protoc. *4*, 44–57. https://doi.org/10.1038/nprot.2008.211.
76. Guzman, S.J., Schlögl, A., and Schmidt-Hieber, C. (2014). Stimfit: quantifying electrophysiological data with Python. Front. Neuroinf. *8*, 16. https://doi.org/10.3389/fninf.2014.00016.
77. Smith, T., Heger, A., and Sudbery, I. (2017). UMI-tools: modeling sequencing errors in Unique Molecular Identifiers to improve quantification accuracy. Genome Res. *27*, 491–499. https://doi.org/10.1101/gr.209601.116.
78. Taniguchi, H., He, M., Wu, P., Kim, S., Paik, R., Sugino, K., Kvitsiani, D., Fu, Y., Lu, J., Lin, Y., et al. (2011). A resource of Cre driver lines for genetic targeting of GABAergic neurons in cerebral cortex. Neuron *71*, 995–1013. https://doi.org/10.1016/j.neuron.2011.07.026.
79. Madisen, L., Zwingman, T.A., Sunkin, S.M., Oh, S.W., Zariwala, H.A., Gu, H., Ng, L.L., Palmiter, R.D., Hawrylycz, M.J., Jones, A.R., et al. (2010). A robust and high-throughput Cre reporting and characterization system for the whole mouse brain. Nat. Neurosci. *13*, 133–140. https://doi.org/10.1038/nn.2467.
80. Tsien, J.Z., Chen, D.F., Gerber, D., Tom, C., Mercer, E.H., Anderson, D.J., Mayford, M., Kandel, E.R., and Tonegawa, S. (1996). Subregion- and cell type-restricted gene knockout in mouse brain. Cell *87*, 1317–1326.
81. Nakazawa, K., Sun, L.D., Quirk, M.C., Rondi-Reig, L., Wilson, M.A., and Tonegawa, S. (2003). Hippocampal CA3 NMDA receptors are crucial for memory acquisition of one-time experience. Neuron *38*, 305–315.
82. Madisen, L., Mao, T., Koch, H., Zhuo, J.M., Berenyi, A., Fujisawa, S., Hsu, Y.W.A., Garcia, A.J., 3rd, Gu, X., Zanella, S., et al. (2012). A toolbox of Cre-dependent optogenetic transgenic mice for light-induced activation and silencing. Nat. Neurosci. *15*, 793–802. https://doi.org/10.1038/nn.3078.
83. Van Nostrand, E.L., Pratt, G.A., Shishkin, A.A., Gelboin-Burkhart, C., Fang, M.Y., Sundararaman, B., Blue, S.M., Nguyen, T.B., Surka, C., Elkins, K., et al. (2016). Robust transcriptome-wide discovery of RNA-binding protein binding sites with enhanced CLIP (eCLIP). Nat. Methods *13*, 508–514. https://doi.org/10.1038/nmeth.3810.
84. Zhang, C., Zhang, Z., Castle, J., Sun, S., Johnson, J., Krainer, A.R., and Zhang, M.Q. (2008). Defining the regulatory network of the tissue-specific splicing factors Fox-1 and Fox-2. Genes Dev. *22*, 2550–2563. https://doi.org/10.1101/gad.1703108.
85. Di Bartolomei, G., and Scheiffele, P. (2022). An optimized protocol for the mapping of cell type-specific ribosome-associated transcript isoforms from small mouse brain regions. Methods Mol. Biol. *2537*, 37–49. https://doi.org/10.1007/978-1-0716-2521-7_3.
86. de la Grange, P., Dutertre, M., Correa, M., and Auboeuf, D. (2007). A new advance in alternative splicing databases: from catalogue to detailed analysis of regulation of expression and function of human alternative splicing variants. BMC Bioinf. *8*, 180. https://doi.org/10.1186/1471-2105-8-180.
87. Noli, L., Capalbo, A., Ogilvie, C., Khalaf, Y., and Ilic, D. (2015). Discordant growth of monozygotic twins starts at the blastocyst stage: a case study. Stem Cell Rep. *5*, 946–953. https://doi.org/10.1016/j.stemcr.2015.10.006.
88. Bischofberger, J., Engel, D., Li, L., Geiger, J.R.P., and Jonas, P. (2006). Patch-clamp recording from mossy fiber terminals in hippocampal slices. Nat. Protoc. *1*, 2075–2081. https://doi.org/10.1038/nprot.2006.312.
89. Barry, P.H. (1994). JPCalc, a software package for calculating liquid junction potential corrections in patch-clamp, intracellular, epithelial and bilayer measurements and for correcting junction potential measurements. J. Neurosci. Methods *51*, 107–116. https://doi.org/10.1016/0165-0270(94)90031-0.

STAR★METHODS

KEY RESOURCES TABLE

REAGENT or RESOURCE	SOURCE	IDENTIFIER
**Antibodies**		
Rabbit anti-SAM68	Iijima et al.[35]	N/A
Rabbit anti-SLM2	Iijima et al.[35]	N/A
Guinea pig polyclonal anti-SLM2	Iijima et al.[35]	N/A
Mouse anti-NeuN	Chemicon	#MAB377
Rat anti-HA monoclonal clone 3F10	Roche	#11867431001
Rabbit anti-Ctip2 polyclonal	Novus Biological	#NB100-2600
Donkey anti-guinea pig IgG Cy5-conjugated	Jackson ImmunoResearch	#706-175-148
Donkey anti-rabbit IgG Cy3-conjugated	Jackson ImmunoResearch	#711-165-152
Donkey anti-guinea pig IgG Cy3-conjugated	Jackson ImmunoResearch	#706-165-148
Donkey anti-rabbit IgG Alexa 488-conjugated	Thermo Fisher	#A-21206
**Chemicals, peptides, and recombinant proteins**		
anti-HA magnetic beads clone 2–2.2.14	Thermo Fisher/Pierce	#88836
Complete mini Protease Inhibitors, EDTA-free	Roche	#04693159001
RNAsin	Promega	#N2115
Cycloheximide	Sigma	#C7698
Heparin	Sigma	#H3393
IGEPAL-CA640 (NP-40)	Sigma	#18896
RNeasy Micro plus kit	Qiagen	#74034
Dynabeads™ Protein A	Invitrogen	#10001D
TURBO™ DNase (2 U/ul)	Invitrogen	#AM2238
Ambion™ RNase I (100 U/ul)	Invitrogen	#AM2295
T4 PNK (10 U/ul)	NEB	#M0314L
T4 RNA Ligase 1 (ssRNA Ligase), High Concentration	NEB	#M0437M
Proteinase K, Molecular Biology Grade (0.8 U/ul)	NEB	#P8107S
Q5® High-Fidelity 2X Master Mix	NEB	#M0492S
ExoSAP-IT™	Applied Biosystems	#78200.200.UL
SuperScript™ III Reverse Transcriptase	Invitrogen	#18080093
Dynabeads™ MyOne™ Silane	Invitrogen	#37002D
NuPAGE™ Transfer Buffer (20X)	Invitrogen	#NP0006
NuPAGE™ 4–12%, Bis-Tris, 1.5 mm, Mini Protein Gels	Invitrogen	#NP0335BOX
NuPAGE™ MOPS SDS Running Buffer (20X)	Invitrogen	#NP0001
Amersham™ Protran® Western blotting membranes, nitrocellulose	Sigma	#GE10600002
RNA Clean & Concentrator-5	Zymo Research	#R1015
Acid-Phenol:Chloroform, pH 4.5	Invitrogen	#AM9720
Phase Lock Gel™ Heavy tubes	5 PRIME	#2302830
Urea for molecular biology	AppliChem	#A1049
Prolong Diamond Antifade Mountant	Life Technologies	#P36970
Dako Fluorescence Mounting medium	Agilent	#S3023
OCT	Tissue-Tek	
**Critical commercial assays**		
HiSeq SBS Kit v4	Illumina	#FC-401-4003
NGS Fragment Analysis Kit	Advanced Analytica	#DNF-473

(*Continued on next page*)

Continued

REAGENT or RESOURCE	SOURCE	IDENTIFIER
RNA 6000 Pico Chip	Agilent	#5067–1513
QuantiFluor RNA System	Promega	#E3310
**Deposited data**		
RNA-seq data	This paper	GEO: GSE209870
CLIP-seq data	This paper	GEO: GSE220062
RNA-seq data *Sam68*^{KO}	Witte et al.[66]	PRIDE: PRJEB27529
RNA-seq data *Slm2*^{KO}	Traunmüller et al.[37]	GEO: GSE79902
**Experimental models: Organisms/strains**		
Mouse: C57BL/6J	Janvier	N/A
Mouse: Grik4-cre	Jackson Laboratories	strain #:006,474
Mouse: Camk2a-cre	Jackson Laboratories	strain #:005,359
Mouse: Sst-IRES-Cre	Jackson Laboratories	strain #:013,044
Mouse: Ai32 ROSA-stop-ChR2/H134R	Jackson Laboratories	strain #:024,109
Mouse: Slm2/KHDRBS3 flox	Traunmüller et al.[36]	N/A
Mouse: Ai9 ROSA-stop-tomato	Jackson Laboratories	strain #:007,909
Mouse: RjOrl:SWISS	Janvier	N/A
Mouse: Rpl22-HA	Jackson Laboratories	strain #:029,977
**Oligonucletides**		
For oligonucleotides, see Table S3	N/A	N/A
**Software and algorithms**		
Fiji	Traunmüller et al.[67]	RRID:SCR_002285
FeatureCounts	Liao et al.[68]	RRID:SCR_012919
CTK	Shah et al.[42]	RRID:SCR_019034
mCross	Feng et al.[44]	N/A
FastQC 0.11.8	N/A	RRID:SCR_014583
Picard v1.119	N/A	RRID:SCR_006525
RSeQC v2.3.9	N/A	RRID:SCR_005275
SAMTOOLS 1.13	Li et al.[69]	RRID:SCR_002105
STAR 2.7.9a	Dobin et al.[70]	RRID:SCR_004463
DESEQ2	Love et al.[71]	RRID:SCR_015687
CLIPper 2.0.0	Yeo et al.[72]	https://github.com/YeoLab/clipper
IDR 2.0.3	Li et al.[73]	RRID:SCR_017237
Sashimi.py	Zhang et al.[74]	https://github.com/ygidtu/sashimi.py
DAVID	Huang et al.[75]	RRID:SCR_001881
SynGO	Koopmans et al.[45]	RRID:SCR_017330
Ethovision10	Noldus	N/A
Stimfit	Guzman et al.[76]	RRID:SCR_016050
pyABF	N/A	http://swharden.com/pyabf
Imaris 7.0.0	Bitplane AG	RRID:SCR_007370
Cutadapt 3.4	N/A	RRID:SCR_011841
UMI-tools 1.1.1	Smith et al.[77]	RRID:SCR_017048

RESOURCE AVAILABILITY

Lead contact

Further information and requests for resources and reagents should be directed to the lead contact, Peter Scheiffele (peter.scheiffele@unibas.ch)

Materials availability

This study did not generate new unique reagents.

Data and code availability

- RNA-seq and CLIP-seq data have been deposited at GEO and are publicly available. Accession numbers are listed in the key resource table. Microscopy data reported in this paper will be shared by the lead contact upon request.
- This paper does not report original code.
- Any additional information required to reanalyze the data reported in this paper is available form the lead contact upon request.

EXPERIMENTAL MODEL AND SUBJECT DETAILS

We used mice in C57BL/6J background obtained from Janvier or mouse lines generated in previous studies.[36] The following transgenic mouse lines were obtained from Jackson Laboratory and maintained in C57BL/6J background: Grik4-cre (strain #:006,474), Sst-IRES-Cre (strain #:013,044), Camk2a-cre (strain #:005,359), Rpl22-HA (strain #:029,977), Ai9 ROSA-stop-tomato (Strain #:007,909), Ai32 ROSA-stop-ChR2/H134R (strain #:024,109), *Slm2* flox (Khdrbs3flox) mice were described in Traunmüller et al.[36] and are available at Jackson Laboratories (strain # strain #:029,273). Male and female mice (similar numbers) were used for acute slice preparations at postnatal day 17–70, for immunohistochemistry at embryonic day 16.5, postnatal day 2–3, and postnatal day 28–56. No influence of sex was determined. Behavioral experiments were conducted exclusively with male mice to reduce potential variability due to estrous cycle stage. Littermates were randomly assigned to experimental groups. All animals were group-housed with free access to food/water in accordance with the animal welfare guidelines and animal experimentation was reviewed and approved by the cantonal veterinary office of Basel-Stadt, Switzerland.

METHOD DETAILS

Mice

All procedures involving animals were approved by and performed in accordance with the guidelines of the Kantonales Veterinäramt Basel-Stadt.

Slm2 floxed mice have been generated in the Scheiffele laboratory and were previously described.[36] *Rpl22-HA* (RiboTag) mice,[49] *SST-cre* mice,[78] Ai9 tdTomato,[79] *CamK2-cre* mice,[80] *Grik4-cre* mice,[81] ChR2-flox mice[82] were obtained from Jackson Laboratories (Jax stock no: 011,029, 013,044, 007,905, 005,359, 006,474, 012,569 respectively). All lines were maintained on a C57Bl6/J background. The specificity of cre-lines for recombination of the Rpl22-allele and/or $Slm2^{flox}$ was confirmed by immunohistochemistry. *Grik4-cre* mice require particular attention due to high rate of spontaneous germline recombination.

Antibodies

Polyclonal antibodies for SLM2 and SAM68 were previously described.[35] Additional antibodies are rat anti-HA (Roche, #11867431001, 1:1000), mouse anti-NeuN (Chemicon #MAB377 1:2000), and rabbit anti-CTIP2 (Novus Biologicals, #NB100-2600). Secondary antibodies included donkey anti-rat IgG-Cy3 and Cy5 (Jackson ImmunoResearch, 712-165-153, 706-175-148 1:1000). DAPI nuclear stain was co-applied with secondary antibody at a final concentration of 0.5 μg/mL.

Primary hippocampal cell culture

Primary hippocampal culture was prepared from RjOrl:SWISS E16 mouse embryos. Hippocampi were dissected in plain DMEM (Invitrogen), minced and transferred in 2mL DMEM to a 15mL tube. 2mL of the 0.25% to the final concentration of 0.125% were added and incubated for 25 min at 37°C in a water bath. Then 100μL of 1 mg/mL DNaseI (Roche) were added and incubated for additional 5min. The digestion solution was removed and hippocampi pieces were washed twice with DMEM containing 10% fetal bovine serum. Subsequently hippocampi pieces were triturated in 1mL neurobasal medium supplemented with B27 (Invitrogen), Glutamax (Invitrogen) and penicillin/streptomycin (Invitrogen). After trituration 4mL of neurobasal medium was added, cell suspension was filtered through a 70μL strainer and centrifuged for 10 min at 100xg. Supernatant was removed and cell pellet was resuspended in 5mL neurobasal medium. 100.000–120.000 cells per well were plated in a 24 well plate with cover slips coated with poly-D-lysine and laminin.

Immunohistochemistry, image acquisition and statistical analysis

Cultured cells were fixed at day in vitro (DIV) 12 with 4% PFA in 1× PBS for 10 min at RT and washed 3× with 1xPBS. Cells were stained for endogenous Sam68 and SLM2 with polyclonal antibodies as previously described in Iijima et al., 2014. Briefly, fixed cells were blocked in blocking solution (5% milk, 0.1% Triton X-100 in PBS) for 1h at RT and then incubated with the primary antibodies anti-SLM2 (1:4000) and anti-SAM68 (1:2000) in blocking solution overnight at 4°C. After 3× washes with PBS cells were incubated for 1h with anti-rabbit-Cy3 and anti-guinea pig Cy5 antibodies in blocking buffer at RT, washed, stained with DAPI and mounted on glass slides. Images were acquired on an inverted LSM880 confocal microscope (Zeiss) using 63× Apochromat objectives in super-resolution Airyscan mode.

For immunohistochemistry on brain sections, postnatal animals (male and female) were transcardially perfused with fixative (4% paraformaldehyde in 100mM phosphate buffer, pH 7.2) and post-fixed over night at 4°. Embryonic brains were drop-fixed for 24h.

Brain samples were immersed in 30% sucrose in 1X PBS for 48h, cryoprotected with Tissue-Tek optimum cutting temperature (OCT) and frozen at -80°. Early postnatal, adolescent and adult tissue was sectioned at 40μm on a cryostat and collected in 1X PBS, whereas embryonic tissue was sectioned at 20μm and collected directly on glass slides. Floating sections were blocked for 1h at RT in 10% Normal Donkey Serum +0.05% Triton X-100, immunostained over night at 4°C with primary antibody incubation diluted in 10% Normal Donkey Serum +0.05% Triton X-100. On slide staining was performed with blocking in 5% Normal Donkey Serum +3% BSA +0.05% Triton X-100 for 1h at RT, followed by RT incubation of primary antibody diluted in 1% Normal Donkey serum for 36h. Secondary antibodies were diluted in 1X PBS +0.05% Triton X-100 for 2h at RT for protein detection, except for on slide staining for which antibodies were diluted in 1X Normal Donkey Serum for 1.5 h at RT. Sections were mounted on glass slides with Prolong Diamond Antifade Mountant or Dako Fluorescence Mounting medium. Images were acquired at room temperature on an upright LSM700 confocal microscope (Zeiss) using 40× Apochromat objectives controlled by Zen 2010 software (1μm z-stacks). Hippocampal overview images were generated at room temperature on a Slidescanner AxioScan.Z1 (Zeiss) using a 10× objective. Stacks of 24μm thickness (4μm intervals) were used for a maximum intensity projection. Overview images of embryonic and P2 animals were taken on an LSM700 upright using a 10× Apochromat objective controlled by Zen 2010 software and image tiling. Images were analyzed and assembled using ImageJ (Fiji) and Adobe Illustrator software.

SLM2 intensity levels were characterized in NeuN + cells residing in either CA1, CA3 or DG regions of the hippocampus. SST + neurons were identified by genetic labeling using SST-cre mice crossed with tdTomato. Intensity levels were determined using in three dimensions using Imaris 7.0.0, Bitplane AG). Three dimensional surfaces were created around each nucleus of either cell class and the labeling intensity was automatically generated by the software based on the intensities of isolated pixels (determined as arbitrary units).

SLM2 knock-out efficiency was determined by comparing WT and SLM2 conditional mutants in either CA1 (Camk2), CA3 (Grik4) or the stratum oriens above CA1 for SST. Intensity levels for calling a neuron SLM2+ or SLM2-were previously determined by the intensity levels of SLM2 observed in the dentate gyrus of the same section. Following this, the number of SLM2+ and SLM2-neurons in the respective area imaged with 40× was quantified. For quantification in CA1 and CA3 mice were 5–6 weeks of age whereas quantification in tdTomato+, SST + neurons was performed at p28. This strategy had been used as CamK2- and Grik4-cre recombinases are expressed at later developmental stages.

Quantification of the percentage of either SLM2 or HA + cells was performed as follows: Within an area of either CA1 or CA3 of mice expressing Rpl22, the number of HA + cells co-expressing SLM2 were determined. From the same image the number of SLM2+ cells that did or did not co-label with HA were additionally determined.

eCLIP library preparation

The CLIP experiments, library preparation, and oligonucleotides were performed essentially as described by Nostrand et al.[83] There were minor modifications to the detailed method, briefly described here: Mouse whole brains or hippocampi were rapidly dissected on ice and immediately flash frozen in liquid nitrogen. The brain samples were ground on dry ice first in a custom-made metal grinder and a porcelain mortar. The frozen powder was transferred into a plastic Petri dish (10 or 6cm diameter) and distributed in a thin layer. Samples were UV-crosslinked 3× with 400 mJ/cm^2 on dry ice with a UV-crosslinker (Cleaver Scientific) with mixing and redistributing of the powder between single UV exposures. The crosslinked powder was re-suspended in 10mL (for 1 × whole brain) or 4.5mL (for 4 hippocampi) of the CLIP-lysis buffer (50mM Tris-HCl pH 7.5, 100mM NaCl, 1% NP-40, 0.1% SDS, 0.5% sodium deoxycholate) supplemented with 1 tablet per 10mL buffer of the protease inhibitors (Roche) and 4U per ml buffer Turbo-DNase (Thermofisher), transferred into a glass homogenizer and homogenized by 30 strokes on ice. 1mL aliquots were transferred to 2mL tubes, 10μL of RNaseI (Thermofisher) diluted in PBS (1:5 or 1:25) were added to a 1mL aliquot. Samples were incubated at 37°C with shaking (1′200 × rpm) for 5 min and put on ice. 10μL RNasin RNase-inhibitor (40U/l, Promega) were added, samples were mixed and centrifuged for 15 min at 16′000 × g, 4°C. After centrifugation the supernatants were transferred to a new tube and 60μL from each sample were taken for sized matched INPUT (SMIn). To the rest 1ug/mL of affinity purified anti-SLM2 antibody was added and samples were incubated for 2h at 4°C in an overhead shaker. Then 10μL of Protein-A magnetic beads (Thermofisher) per 1μg antibody were added to each sample and samples were incubated for additional 2h at 4°C in an overhead shaker. Beads were washed 2× with the high salt wash buffer (50mM Tris-HCl pH7.5, 1M NaCl, 1mM EDTA, 1% NP-40, 0.1% SDS, 0.5% sodium deoxycholate), 2× CLIP-lysis buffer, 2× with low salt wash buffer (20mM Tris-HCl pH7.5, 10mM $MgCl_2$, 0.2% Tween 20) and 1× with PNK buffer 70mM Tris-HCl pH6.5, 10mM $MgCl_2$). Beads were re-suspended in 100μL PNK-mix (70mM Tris-HCl pH6.5, 10mM $MgCl_2$, 1mM DTT, 100U RNasin, 1U TurboDNase, 25U Polynucleotide-Kinase) and incubated for 20 min at 37°C in a thermomixer with shaking (1200 × rpm). After RNA dephosphorylation beads were washed as before with 2× high salt, 2× lysis and 2× low salt buffers and additionally with 1× Ligase buffer (50mM Tris-HCl pH7.5, 10mM $MgCl_2$). Beads were re-suspended in 50μL ligase mix (50mM Tris-HCl pH7.5, 10mM $MgCl_2$, 1mM ATP, 3% DMSO, 15% PEG8000, 30U RNasin, 75U T4 RNA-ligase). 10μL of the beads/ligase mix were transferred to a new tube and 1μL of pCp-Biotin (Jena Bioscience) were added to validate IP of the RNA-protein-complexes by Western blot. To the rest (40μL) 4μL of the RNA-adaptor mix containing 40μM of each RNA_X1A & RNA_X1B (IDT) were added and samples were incubated for 2h at RT. After adaptor ligation samples were washed 2× with high salt, 2× with lysis and 1× with low salt buffers. Beads were re-suspended in 1× LDS sample buffer (Thermofisher) supplemented with 10 μM DTT and incubated at 65°C for 10min with shaking at 1200 × rpm. Eluates or inputs were loaded on 4–12% Bis-Tris, 10-well, 1.5mm gel (Thermofisher) and separated at 130V for ca. 1.5h. Proteins were transferred to the nitrocellulose membrane (Amersham) overnight at 30V. After transfer the membranes were placed in a

15cm Petri dish on ice and an area between 55 and 145kDa was cut out in small pieces and transferred to 2mL tube. For CLIP samples RNA extraction, reverse transcription using AR17 primer, cDNA clean-up using silane beads (Thermofisher), second adaptor ligation (rand103Tr3) and final cDNA purification were performed as previously described.[83] For sized matched input samples (SMIn) isolated RNA was dephosphorylated.

The sequencing libraries were amplified using Q5-DNA polymerase (NEB) and i50X/i70X Illumina indexing primers (IDT). 14 cycles were used for the amplification of whole brain libraries and 16 cycles for hippocampus libraries. Corresponding SMIn libraries were amplified with 12 cycles for whole brain and 16 cycle for hippocampus samples. The amplified libraries were purified and concentrated first with ProNEX size selective purification system (Promega) using sample/beads ratio of 1/2.4. The purified libraries were loaded on a 2% agarose gel, the area corresponding to the size between 175bp and 350bp was cut and the libraries were extracted from the gel using gel extraction kit (Machery&Nagel) and eluted with 16μL.

Concentrations and size distributions of the libraries were determined on Fragment analyzer system (Agilent). 75bp paired-end sequencing was performed on the NextSeq500 platform using Mid Output Kit v2.5 (150 cycles). Adaptor and primer sequences used in this study are listed in Table S3.

eCLIP data processing

The raw reads were processed to obtain unique CLIP tags mapped to mm10 using CTK,[42] as described previously.[44] Unique tags from replicates were combined for all analyses. Significant CLIP tag clusters were called by requiring $p < 0.05$ after Bonferroni multiple-test correction. Crosslinking-induced truncation sites (CITS) were called by requiring FDR<0.001. We performed 7-mer enrichment analysis using significant peaks with peak height (PH) $\geq$ 10 tags. Peaks were extended for 50 nt on both sides relative to the center of the peak to extract the foreground sequences. Background sequences were extracted from the flanking regions of the same size (-550, -450) and (450, 550) relative to the peak center. Sequences with more than 20% of nucleotides overlapping with repeat masked regions were discarded. 7-mers were counted in repeat-masked foreground and background sequences, and the enrichment of each 7-mer in the foreground relative to the background was evaluated using a binomial test, from which *Z* score (and a p value) was derived. In parallel, eCLIP data was analyzed following the ENCODE pipeline using the CLIPper peak calling tool (https://github.com/YeoLab/clipper; https://github.com/YeoLab/eclip), followed by IDR (https://github.com/nboley/idr) to identify peaks reproducibly identified between replicates. Both analysis pipelines (CTK and CLIPper) gave similar results and led to the same biological conclusions.

Motif analysis

UWAA motif sits were searched in genic regions and their conservation was evaluated using branch length score (BLS) estimated from multiple alignments of 40 mammalian species.[84] *De novo* motif discovery was performed using mCross, an algorithm that augments the standard PWM model by jointly modeling RBP sequence specificity and the precise protein-RNA crosslink sites at specific motif positions at single-nucleotide resolution.[44] For this analysis, the top 10 enriched 7-mers from significant peak regions were used as seed to search for overrepresented motifs around CITS sites, as described previously.[44] The motif with the maximum motif scores was chosen as the represented motif.

Gene ontology analysis

Gene Ontology was performed using the DAVID functional annotation (cellular compartment) online tool (https://david.ncifcrf.gov/tools.jsp). The input list for the background was the list of all genes detected in a hippocampal sample analyzed by bulk RNA sequencing.[37] Genes that had significant peak expression in the CLIP dataset either for hippocampus or whole brain samples were used. The top ten significant (Benjamini Hochberg for p value correction), and non-redundant, terms were displayed in Figure 1G (whole brain) and Figure S1E (hippocampus).

RiboTRAP pulldowns, RNA purification and quality control

RiboTRAP purifications were performed as previously described.[21,85] For CamK2 and Grik4 pull downs animals were between postnatal day 39–42, for SST neurons between postnatal day 28–30. Mice were anesthesized with isoflurane and following cervical dislocation hippocampal tissue was rapidly dissected in ice-cold PBS and lysed in 0.5mL for single animals (Camk2 and Grik4) and 1mL for pools of two animals (SST) in homogenization buffer containing 100mM KCl, 50mM Tris-HCl pH 7.4, 12mM $MgCl_2$, 100 μg/mL cycloheximide (Sigma-Aldrich # 66-81-9), 1 mg/mL heparin (Sigma-Aldrich #H339350KU), 1× complete mini, EDTA-*free* protease inhibitor cocktail (Roche #11836170001), 200 units/mL RNasin plus inhibitor (Promega #N2618) and 1mM DTT (Sigma-Aldrich #3483-12-3). The lysate was centrifuged for 10 min at 2.000xg, 1% final concentration of Igepal-CA630 (Sigma Aldrich #18896) was added to the supernatant and incubated on ice for 5min, followed by an additional spin at 12.000xg. 1% of input was saved in RLTplus buffer (Qiagen RNeasy Micro Kit #74034) supplemented with 2-Mercaptoethanol before 20μL or 15μL of HA-magnetic beads (Pierce, #88837) were added to the excitatory or inhibitory pull down, respectively. Lysate/bead mixtures were incubated at 4° for 3-4hours under gentle rotation and were afterward washed 4 times with wash buffer containing 300mM KCl, 1% Igepal-CA630, 50mM Tris-HCl, pH7,4, 12mM $MgCl_2$, 100 μg/mL Cycloheximide and 1mM DTT. RNA was eluted from beads with 350μL RLT plus buffer supplemented with 2-Mercaptoethanol as per manufacturers instructions.

RNA of input and RiboTrap IP samples was purified using the RNeasy Plus Micro Kit (Qiagen #74034) following manufacturer's instructions. RNA was further analyzed using an RNA 6000 Pico Chip (Agilent, 5067–1513) on a Bioanalyzer instrument (Agilent Technologies) and only RNA with an integrity number higher than 7.5 was used for further analysis. RNA concentration was determined by Fluorometry using the QuantiFluor RNA System (Promega #E3310) and 20ng of RNA was reverse transcribed for analysis of marker enrichment by quantitative PCR. Only samples which had an enrichment for hippocampal layer specific excitatory neuron markers and a de-enrichment for inhibitory or glia markers were further used for CamK2 and Grik4. SST pulldowns exhibited an enrichment in inhibitory neuron markers and a de-enrichment in excitatory and glia markers.

Library preparation and illumina sequencing

Four biological replicates per cell class and genotype were further analyzed. Library preparation was performed with 50ng of RNA using the TruSeq PolyA + Stranded mRNA Library Prep Kit High Throughput (Illumina, RS-122-2103). Libraries were quality-checked on a Fragment Analyzer (Advanced Analytical) using the Standard Sensitivity NGS Fragment Analysis Kit (Advanced Analytica, DNF-473), revealing high quality of libraries (average concentration was 49 ± 14 nmol/L and average library size was 329 ± 8 base pairs). All samples were pooled to equal molarity and the pool was quantified by PicoGreen Fluorometric measurement. The pool was adjusted to 10pM for clustering on C-Bot (Illumina) and then sequenced Paired-End 101 bases using the HiSeq SBS Kit v4 (Illumina, FC-401-4003) on a HiSeq 2500 system. Primary data analysis was performed with the Illumina RTA version 1.18.66.3 and bcl2fastq-v2.20.0.422.

Quality control and RNA-seq pre-processing

The gene expression and alternative splicing analysis of the RNA-Sequencing data were performed by GenoSplice technology (www.genosplice.com) and have been additionally described in.[21] Data quality, reads repartition (e.g., for potential ribosomal contamination), and insert size estimation were performed using FastQC v0.11.8, Picard-Tools v1.119, Samtools 1.13 and rseqc v2.3.9. This first quality check identified one sample in the pool of DCamK2 which displayed an accumulation of reads on the 3′end and displayed higher ribosomal contamination. Thus, this sample was excluded from further analyses. Reads were mapped using STARv2.4.0[70] on the mm10 Mouse genome assembly. Reads were mapped using STARv2.4.0[70] against the exons defined in the proprietary Mouse FAST DB v2016_1 database,[86] using a mismatch cutoff of 2 and discarding reads with 10 or more alignments. The minimum chimeric segment length was 15. Read counts were summarized using featureCounts[68] in two stages. First, unique reads per exon were counted. In the second stage, multimapping reads were fractionally allocated to exons based on the distribution of unique counts of exons within a gene. Total counts were then calculated based on three constitutivity classes defined in FAST DB: class 2 includes exons present in more than 75% of annotated transcripts for a gene ("constitutive"), class 1 includes exons present in 50–75% of transcripts ("semi-constitutive"), and class 0 includes exons present in less than 50% of transcripts ("alternative"). Total counts per gene were summed from constitutivity class 2 exons if their FPKM values exceed 96% of the background FPKM based on intergenic regions. If counts from class 2 exons were insufficient to exceed the detection threshold, class 1 and eventually class 0 exon counts were included to reach the detection threshold.

Differential gene expression analysis

Differential regulation of gene expression was performed as described.[87] Briefly, for each gene present in the proprietary Mouse FAST DB v2016_1 annotations, reads aligning on constitutive exons of the gene are counted. Based on these read counts, normalization and differential gene expression are performed using DESEq2.[71] Background expression was defined by reads aligning to intergenic regions, thus, only genes are considered as expressed if their RPKM value (reads per kilo base of transcript per million mapped reads) is greater than 96% of the background RPKM value based on intergenic regions. Only genes expressed in at least 3 out of 4 biological replicates for Grik4 and SST; and in at least 2 out of 3 biological replicates for CamK2 were further analyzed. For all expressed genes, DESeq2 values were generated (values were normalized by the total number of mapped reads of all samples). Fold change in gene expression was calculated by pairwise comparisons, comparing the normalized expression value in the respective WT condition to the corresponding ΔSLM2 condition and p value (unpaired Student's t-test) and adjusted p value (Benjamin and Hochberg) were calculated. Results were considered significantly different for adjusted p values ≤ 0.05 and fold changes $\geq \pm 1.5$.

Alternative splicing analysis

Identification of alternatively spliced exons was performed with two analysis approaches as previously described[21]: "**exon**" and "**pattern**" analysis. The exon analysis takes reads mapping to exonic regions and to exon-exon junctions into account. When reads map onto exon-exon junctions, the reads were assigned to both exons and the minimum number of nucleotides is 7 in order that a read is considered mapped to an exon. An exon was considered to be expressed if the FPKM value (Fragments per kilobase of transcript per million mapped reads) was greater than 96% of the background FPKM value based on intergenic regions. Only exons that were expressed in at least 3 out of 4 biological replicates for Grik4 and SST; and in at least 2 out of 3 biological replicates for CamK2 were further analyzed. Furthermore, for every expressed exon a splicing index (SI) was calculated: This is the ratio between read density on the exon of interest (=number of reads on the exon/exon length in nucleotides) and read density on constitutive exons of the same gene (with constitutive exons defined in FAST DB). The second type of alternative splicing analysis is the Pattern analysis. This type of analysis is taking known splicing patterns annotated in the FAST DB database into account.[86] For each gene all annotated

splicing patterns are defined and an SI is generated by comparing the normalized read density to the alternative annotated patterns.

The Log2 fold change (FC) and p value (unpaired Student's t-test) for both the exon and pattern analysis was calculated by pairwise comparisons of the respective SI values. Results were considered significantly different for p values $\leq$0.01 and log2(FC) $\geq$1 or $\leq$ -1.

Sashimi plots were generated with Sashimi.py.[74]

qPCR analysis for alternative exon usage of *Nrxns* at AS4

Ribotag purified material was reverse transcribed and quantitative PCR was performed. qPCRs were performed on a StepOnePlus qPCR system (Applied Biosystems). Assays were used with a TagMan Master Mix (Applied Biosystems) and comparative C_T method. mRNA levels were normalized to the amount of *Gapdh* cDNA present in the same sample.

Custom gene expression assays were from Applied Biosystems and are described in.[35]

Electrophysiology

Slice preparation

Adult mice (P56-70) were anesthetized with isoflurane (4% in O_2, Vapor, Draeger) or with intraperitoneal injection of ketamine/xylazine (100 mg/kg and 10 mg/kg), and killed by decapitation, in accordance with national and institutional guidelines. For recordings in SST interneurons P17-18 animals were used. Slices were cut as previously described.[88] Briefly, the brain was dissected in ice-cold sucrose-based solution at about 4°C. Horizontal 300- to 350-μm-thick hippocampal brain slices were cut at an angle of about 20° to the dorsal surface of the brain along the dorso−ventral axes of the hippocampus using a Leica VT1200 vibratome. For cutting and storage, a sucrose-based solution was used, containing 87 mM NaCl, 25 mM $NaHCO_3$, 2.5 mM KCl, 1.25 mM NaH_2PO_4, 75 mM sucrose, 0.5 mM $CaCl_2$, 7 mM $MgCl_2$ and 10 mM glucose or 10 mM dextrose (equilibrated with 95% O_2/5% CO_2). Some slices were prepared with additional 1–5 mM ascorbic acid and/or 3 mM pyruvic acid. Slices were kept at 32–35°C for 30 min after slicing and subsequently stored at room temperature either in cutting solution or in artificial cerebrospinal fluid (ACSF): 124mM NaCl, 2.5 mM KCl, 1.25 mM NaH_2PO_4, 2 mM $CaCl_2$, 1–2 mM $MgSO_4$, 26 mM $NaHCO_3$, 10 mM dextrose or 10 mM glucose until experiments were performed at 21 to 22°C. For experiments, slices were, transferred to the recording chamber and perfused (1.5–2.0 mL/min) with oxygenated ACSF at room temperature.

Whole-cell voltage-clamp recordings of EPSCs in CA1 pyramidal neurons

Hippocampal CA1 pyramidal neurons were visually identified in the pyramidal cell layer using Dodt-contrast video microscopy. Somatic whole-cell recordings were made from CA1 pyramidal neurons, which were voltage clamped with a Multiclamp 700B amplifier, and currents were digitized by Digidata 1440a. Patch pipettes (4–8 MΩ) were filled with voltage-clamp solution for excitation response curves: 125mM Cs-gluconate, 2 mM CsCl, 5 mM TEA-Cl, 4 mM ATP, 0.3 mM GTP, 10 mM phosphocreatine, 10 mM HEPES, 0.5 mM EGTA, and 3.5 mM QX-314. Data were filtered at 2 kHz, digitized at 10 kHz, and analyzed with Clampfit 10. SC afferents were stimulated with a small glass bipolar electrode prepared from theta glass (Sutter, BT-150-10) and passed once through a Kimwipe to make a 25–50μM opening. Excitation response curves were quantified from the average of the peak from ten evoked EPSCs (0.1Hz) voltage-clamped at −70mV – near the reversal potential for GABAAR-mediated inhibition. Short-term plasticity was induced with five stimuli of equal intensity at 40 Hz and voltage-clamped at −70mV. Data was analyzed with custom software written for this project using Python 3.7 and the pyABF module (http://swharden.com/pyabf). Significance was assessed by a two-way ANOVA for multiple comparisons.

Whole-cell voltage-clamp recordings of IPSCs in CA1 pyramidal neurons

CA1 pyramidal neurons were visually identified in the pyramidal cell layer using infrared differential interference contrast (IR-DIC) video microscopy. Patch-pipettes (2–4.5 MΩ) were filled with a Cs gluconate-based solution containing: 135mM CsGluc, 2mM CsCl, 10mM EGTA, 10mM HEPES, 2mM $MgCl_2$, 2mM Na_2ATP, 2mM TEA-Cl, 5mM QX314 adjusted to pH 7.3 with CsOH.

A diode laser (DL-473, Rapp Optoelectronic) was coupled to the epifluorescent port of the microscope (Zeiss Examiner, equipped with a 63× NA1.0 water immersion objective; Carl Zeiss Microscopy GmbH, Jena, Germany) via fiber optics. The laser was controlled via TTL pulses. For the optogenetic activation of the axon of SST + interneurons, the field of view was shifted to stratum lacunosum moleculare and laser light was applied at intensities of 0.1–3.2 mW for 2 ms. Optogenetically evoked IPSCs were recorded in presence of 25 μM AP5 and 10 μM NBQX. During the assessment of the voltage dependence of optogenetically activated GABA receptors, the series resistance was compensated at 80%. Membrane potentials were corrected offline by the calculated liquid junction potential of −15.7 mV.[89]

Voltage- and current-clamp recordings in SST + OLM interneurons

In slices from SST-Cre x Ai9tdTomato x SLM2^{flox} mice, putative OLM interneurons were visually identified according to their fluorescence, location in stratum oriens close to the alveus and by their morphology with an oval cell body and bipolar morphology oriented in parallel to the alveus. Somatic whole-cell recordings from s.o SST interneurons close to the alveus were clamped with a Multiclamp 700B amplifier (Molecular Devices, Sunnyvale, CA) and identified using epifluorescence microscopy. Signals were low-pass filtered at 2kHz, digitized at 10kHz. For voltage-clamp recordings, patch pipettes used were between 2 and 6 MΩ and filled with either with a solution containing 135mM $CsMeSO_3$, 10mM HEPES, 9mM NaCl, 0.3mM EGTA, 4mM Mg-ATP, 0.3 Na-GTP, 5mM QX-314, 0.1mM Spermine, 303mOsm, pH = 7.3 or 135mM CsGluc, 2mM CsCl, 10mM EGTA, 10mM HEPES, 2mM $MgCl_2$, 2mM Na_2ATP, 2mM TEA-Cl, 5mM QX314 adjusted to pH 7.3 with CsOH. Cells which had a change in series resistance $\geq$20% from start to the end of the experiment, or a series resistance higher than 25 were excluded from the analysis. Membrane resistance, series resistance and capacitance were constantly monitored by a -5mV step at the end of the trace.

To stimulate CA1 pyramidal neuron axon collaterals, the pipettes were placed into the border region between stratum oriens and alveus at a distance of approx. 200–250μM from the recorded neuron, and electrical stimulation was applied at low intensity (10–50 μA, at least 20× every 10s). The minimal first average response amplitude had to be at least 60pA in order to be further analyzed. 100 μM picrotoxin and 1 μM CGP54626 were added to block $GABA_A$-mediated postsynaptic currents and $GABA_B$ signaling, respectively.

Measurements of intrinsic properties were performed in current-clamp I_c with the following internal solution: 135mM K-gluconate, 5mM NaCl, 5mM MgATP, 0.3mM NaGTP, 10mM Phosphocreatine, 10mM HEPES without the addition of blockers.

Data was analyzed was performed offline using the open-source analysis software Stimfit[76] (https://neurodroid.github.io/stimfit) and customized scripts written in Python. The analysis of voltage-clamp data was performed on mean waveforms. Cumulative distribution analysis (in%) was performed in Prism. Amplitudes were analyzed on individual events of every cell, whereas inter-event intervals were calculated based on the frequency of events per 10s sweep.

Drugs

All drugs were stored as aliquots at −20°C. D-AP5 (50 mM; Tocris) was dissolved in water. Picrotoxin was dissolved at 50 mM in ethanol. CGP 54626 hydrochloride (10 mM; Tocris) and NBQX (20 mM; Tocris) were dissolved in DMSO.

Behavioral analysis

Mice used for behavioral experiments were maintained in C57/Bl6J background, male, between 7 and 9 weeks of age and housed under standard laboratory conditions on a 12h light/dark cycle. All tests were carried out during the light cycle, with standard ceiling light and in at least 3 independent trials. All statistical data are mean ± SEM. Every animal was tested in all behavioral assays (battery testing).

Open Field

Mice were individually exposed to a square open field arena (50 × 50 × 30 cm) made of gray plastic for 10min. Velocity (cm/min) and time spent in the center were extracted from a video-based EthoVision10 system (Noldus).

Novel object recognition task

Animals tested in the Open Field arena on the day before the experiment, were exposed to two identical objects (culture dish flask filled with sand) for 5min in the first trial (acquisition). After 1hour, we tested for Short-term memory by 5min exploration of one familiar (flask) and one novel object (Lego block). The time spent investigating the objects, sniffing less than a centimeter from or touching the object, was scored manually. The time mice spent on the objects was excluded (exploration not directed at the object itself). Only mice spending at least 2 s with the objects in total were included in the analysis. Calculation of discrimination ratio: (time spent with novel object – time spent with familiar object)/total time investigating both objects. Distance traveled was extracted from the video-based EthoVison10 system (Noldus). Time and number of grooming or rearing events, and the time spent investigating the objects was scored manually.

Elevated plus maze

Animals were placed in the center of the maze (arms are 35 cm × 6 cm and 74 cm above the ground) facing the closed arms. The time spent on the open arm was measured during the 5 min test. In addition, the number of total entries (open arms and closed arms) were counted manually.

Marble burying

Animals were exposed to 20 identical black marbles distributed equally (4 × 5) in a standard Type II long cage with 5 cm high bedding for 30 min with ceiling light. For a marble to be counted as buried, approximately ≥75% of its area had to be below the bedding material.

QUANTIFICATION AND STATISTICAL ANALYSIS

Quantification of electrophysiological data was performed using stimfit, histology and behavioral data was quantified by an experimenter blinded toward genotype. Statistical analysis for differential gene expression and alternative splicing events of RNA Sequencing experiments was performed in R and adjusted with the Benjamini Hochberg correction. All other statistical analysis was conducted using Prism version 8.0 and 9.0. Data was tested for normality with the Kolmogorov-Smirnov test and similar standard deviation before appropriate t test were chosen for molecular, electrophysiological and behavioral experiments. Paired t-tests were applied for the comparison of interaction time between familiar and novel objects. When assessing changes in the electrophysiological or behavioral properties in which multiple groups were compared, one or two-way ANOVA's with appropriate correction for multiple comparisons (either Šídák's or Tukey's multiple comparisons tests) were performed. Statistical details for each data panel are also described in the figure legends and STAR Methods section.

Cell Reports, Volume *42*

Supplemental information

A cell-type-specific alternative splicing regulator shapes synapse properties in a *trans*-synaptic manner

Lisa Traunmüller, Jan Schulz, Raul Ortiz, Huijuan Feng, Elisabetta Furlanis, Andrea M. Gomez, Dietmar Schreiner, Josef Bischofberger, Chaolin Zhang, and Peter Scheiffele

Supplementary Material

Figure S1 – related to Figure 1

Figure S2 – related to Figure 2

Figure S3 – related to Figure 3

Figure S4 – related to Figure 4

Figure S5 – related to Figure 4

Figure S6 - related to Figure 4

Figure S1

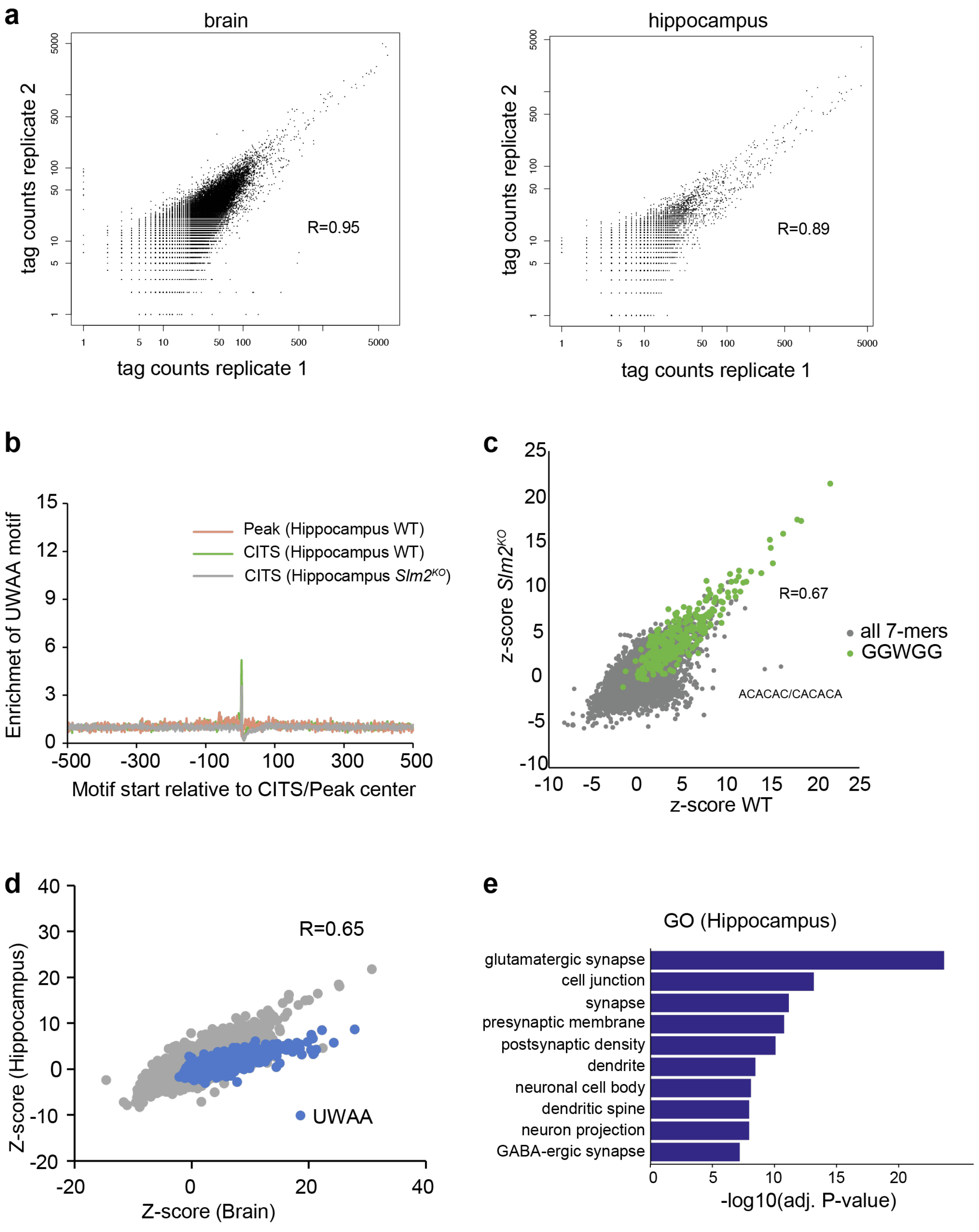

a
brain
tag counts replicate 2
tag counts replicate 1
R=0.95
hippocampus
R=0.89
b
Enrichmet of UWAA motif
Peak (Hippocampus WT)
CITS (Hippocampus WT)
CITS (Hippocampus Slm2KO)
Motif start relative to CITS/Peak center
c
z-score Slm2KO
z-score WT
R=0.67
all 7-mers
GGWGG
ACACAC/CACACA
d
Z-score (Hippocampus)
Z-score (Brain)
R=0.65
UWAA
e
GO (Hippocampus)
glutamatergic synapse
cell junction
synapse
presynaptic membrane
postsynaptic density
dendrite
neuronal cell body
dendritic spine
neuron projection
GABA-ergic synapse
-log10(adj. P-value)

Figure S1. Mapping SLM2 binding sites by eCLIP

a, Correlation plot of tag numbers over called eCLIP tag clusters in replicate 1 (x-axis) and replicate 2 (y-axis) of whole brain (left panel) and hippocampus (right panel) eCLIP data. Pearson's correlation coefficient is shown. **b,** Enrichment of UWAA around CITS is calculated from the frequency of UWAA starting at each position relative to the inferred crosslink sites, normalized by the frequency of the element in flanking sequences in hippocampus eCLIP data from wild-type and *Slm2*^{KO} hippocampus. Enrichment of UWAA around the CLIP tag cluster peak center is shown for comparison. **c,** Correlation plot of 7mer enrichment z-scores from WT and *Slm2*^{KO} hippocampal eCLIP data. The GGWGG motif identified in hippocampal WT eCLIP samples (highlighted in green) is found to the similar extent in global SLM2 knock-out control samples. Pearson's correlation coefficient is shown. **d,** Correlation of 7-mer enrichment z-scores of 100nt region around peak center from whole brain (x-axis) and hippocampus (y-axis) eCLIP data. 7-mers including UWAA are highlighted in blue. Pearson's correlation coefficient is shown. **e,** Gene Ontology analysis (DAVID tools) of genes with SLM2 binding sites in hippocampal eCLIP data identified by CLIPper/IDR. Top 10 enriched gene ontology categories for cellular compartment are displayed.

Figure S2

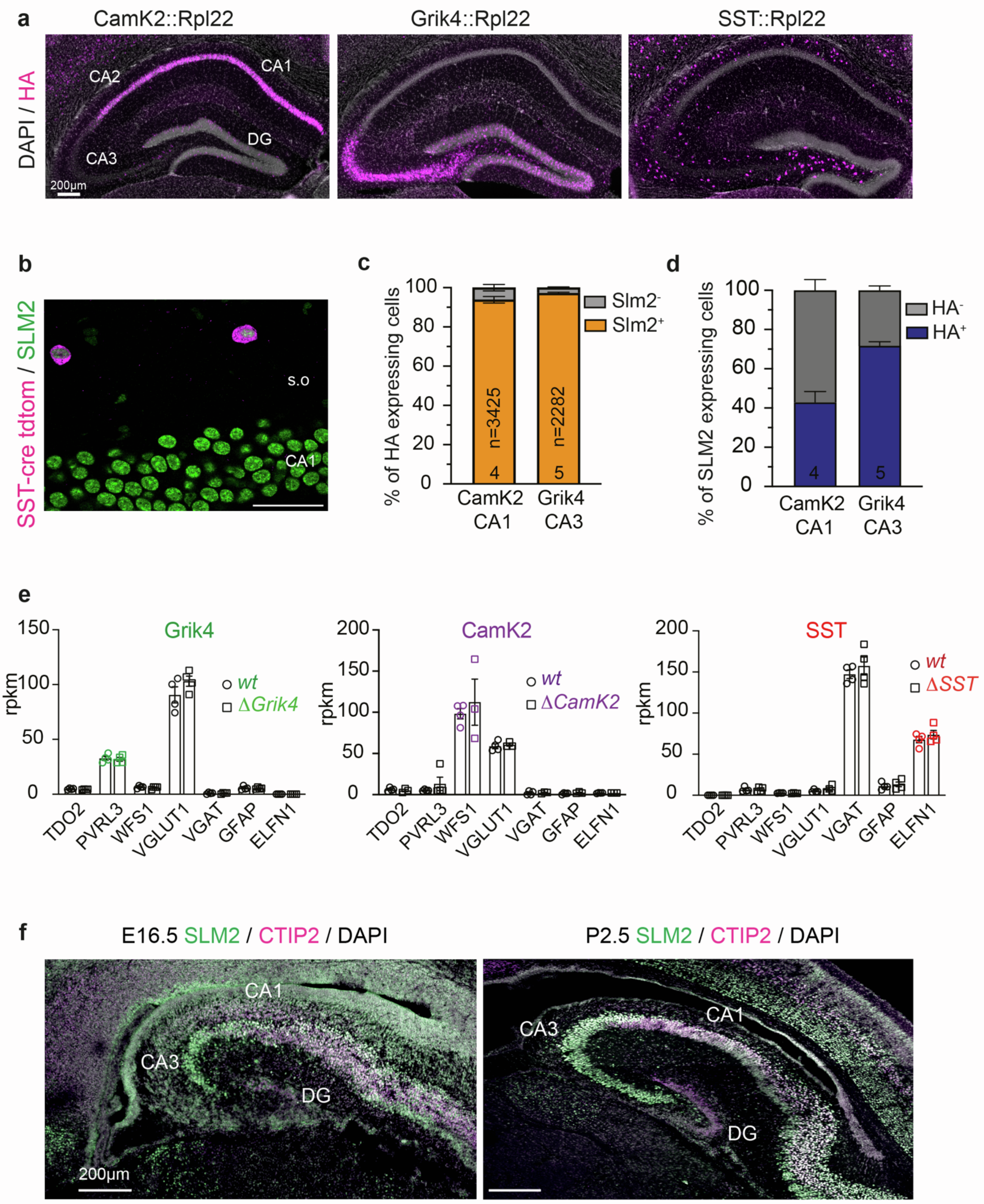
a
CamK2::Rpl22
Grik4::Rpl22
SST::Rpl22
DAPI / HA
CA2
CA1
CA3
DG
200µm
b
SST-cre tdtom / SLM2
s.o
CA1
c
% of HA expressing cells
Slm2-
Slm2+
n=3425
n=2282
4
5
CamK2 CA1
Grik4 CA3
d
% of SLM2 expressing cells
HA-
HA+
e
Grik4
CamK2
SST
rpkm
wt
ΔGrik4
ΔCamK2
ΔSST
TDO2
PVRL3
WFS1
VGLUT1
VGAT
GFAP
ELFN1
f
E16.5 SLM2 / CTIP2 / DAPI
P2.5 SLM2 / CTIP2 / DAPI
CA1
CA3
DG
200µm

Figure S2. Expression and conditional knock-out of SLM2 in the mouse hippocampus.

a, Representative images of cre-dependent expression of HA-tagged ribosomal protein L2 (Rpl22) in CA1 (CamK2::Rpl22), CA3 (Grik4::Rpl22) or SST+ interneurons (SST::Rpl22). Scale bar 200µm, DAPI (grey), HA (magenta). **b,** SLM2 (green) expression in CA1 pyramidal neurons and genetically marked SST+ interneurons (magenta) in the stratum oriens (s.o). Scale bar 40µm. **c,** Quantification of percentages of HA+ neurons defined by either CamK2 or Grik4 cre-recombinase which express SLM2 (SLM2+, orange). CamK2-cre: N=4 animals, n=3425 cells. Grik4-cre: N=5 animals, n=2282 cells. Mean of each replicate ± SEM. **d,** Quantification of SLM2+ neurons in either CA1 or CA3 layers which express Rpl22-HA (HA+, blue). Same images and numbers as for (c). Mean of each replicate ± SEM. **e,** Reads per kilobase million (rpkm) of cell class-specific marker genes in all analyzed cell types and individual replicates of *wt* and ∆*SLM2* animals (N=4). *TDO2*: DG marker, *PVRL3*: CA3, *RGS12*: CA2, *WFS1*: CA1, *VGLUT1*: excitatory neurons, *VGAT*: inhibitory neurons, *GFAP*: glia, *ELFN1*: SST neurons. **f,** Hippocampal tissue from mice immunostained for SLM2 (green), CTIP2 (purple) and DAPI (grey) at E16.5 and P2.5. This demonstrates selective expression of SLM2 in CA1 and CA3 but not dentate granule cells at early stages of hippocampal development. Scale bar is 200µm.

Figure S3

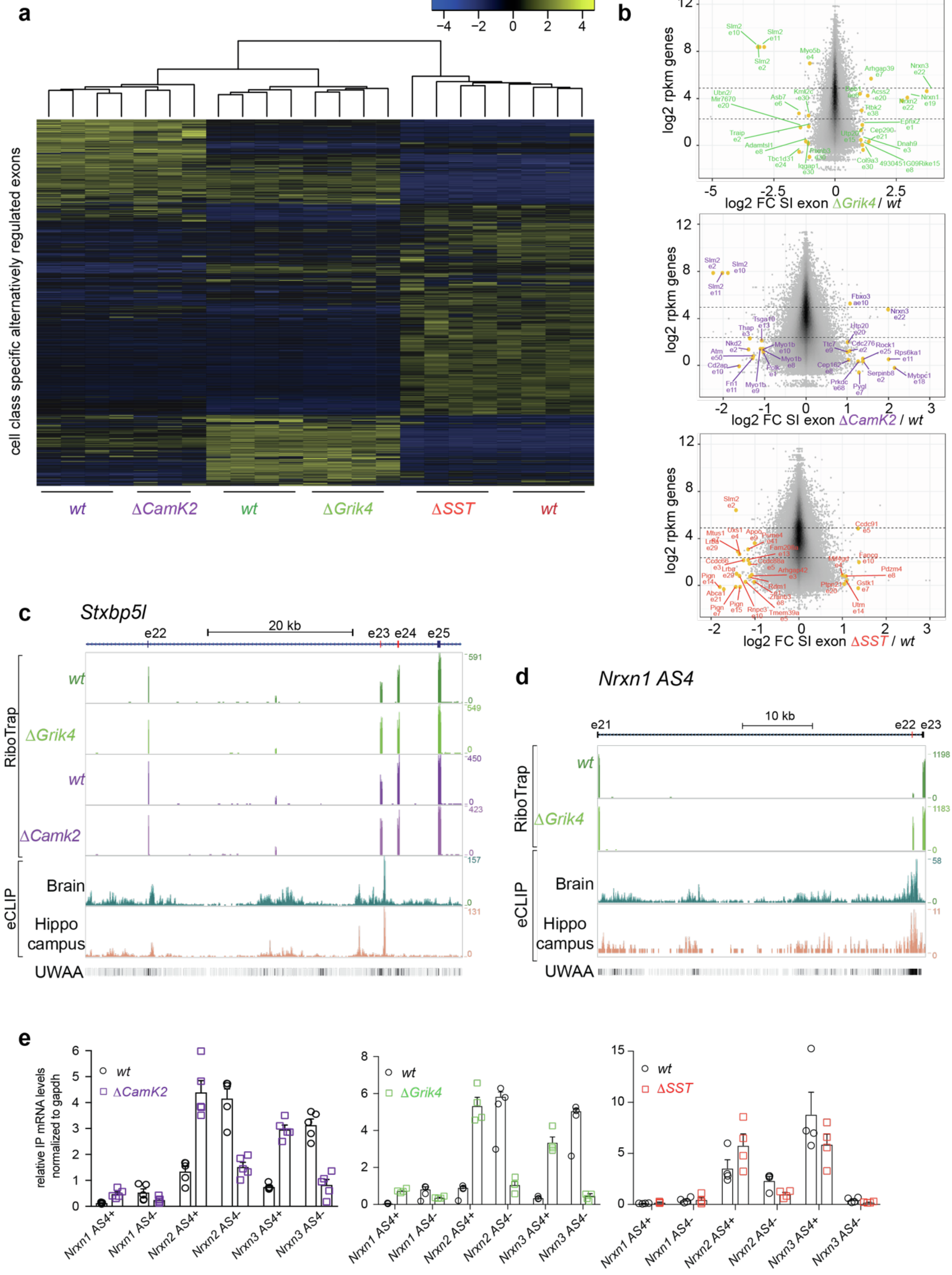
a
Column Z−Score
cell class specific alternatively regulated exons
wt
ΔCamK2
wt
ΔGrik4
ΔSST
wt
b
log2 rpkm genes
log2 FC SI exon ΔGrik4 / wt
log2 FC SI exon ΔCamK2 / wt
log2 FC SI exon ΔSST / wt
c
Stxbp5l
20 kb
e22
e23 e24
e25
RiboTrap
eCLIP
Brain
Hippo campus
UWAA
d
Nrxn1 AS4
10 kb
e21
e22 e23
e
relative IP mRNA levels normalized to gapdh
Nrxn1 AS4+
Nrxn1 AS4-
Nrxn2 AS4+
Nrxn2 AS4-
Nrxn3 AS4+
Nrxn3 AS4-

Figure S3. Alternative splicing in *Slm2* conditional knock-out cells.

a, Heatmap of splicing indices (SI) of exons across individual replicates of *wt* and *Slm2* conditional mutants. Analyzed exons were defined by previously identified, cell class-specific alternative exons[1]. Splicing indices were normalized by row and column. Detailed z-score values, gene and exon names are provided in Table S2. **b,** Log2 fold change SI (splicing index) and p-values for all detected exons (grey). Exons which are significantly differentially regulated by SLM2 in each cell class (called by exon analysis) are marked in yellow. **b,** Log2 fold change of SI of all detected exons (grey) and log2 rpkm values of the corresponding gene. Differentially regulated exons called by the exon analysis are marked in yellow. Gene names and exons involved in the splicing regulation are indicated in purple for differential changes in CamK2, green for Grik4 and red for SST. **c,d,** Integration of RiboTrap and eCLIP analysis for significantly de-regulated exons of *Stxbp5l* (c) and *Nrxn1* (d). SLM2 binding sites in the downstream introns and enrichment of the UWAA binding motif of the Grik4 comparison are illustrated. **e,** Quantitative PCR for alterations in *Nrxn* splicing at the alternatively spliced segment 4 (AS4). Relative *Gapdh* normalized mRNA levels of RiboTRAP IP samples in WT and ∆SLM2 samples. For all PCRs: *wt* CamK2 and ∆*CamK2*: N=5; *wt* Grik4 N=4 and ∆*Grik4* N=5, *wt* SST N=4 and ∆*SST* N=4, except for $Nrxn1^{AS4+}$ N=3. *wt* CamK2 vs ∆*CamK2:* $Nrxn1^{AS4-}$ p= 0.861, $Nrxn1^{AS4+}$ p=0.0019, $Nrxn2^{AS4-}$ p=0.0002, $Nrxn\ 2^{AS4+}$ p=0.0003, $Nrxn3^{AS4-}$ p<*0.0001,* $Nrxn\ 3^{AS4+}$ p<0.0001*; wt* Grik4 vs ∆*Grik4* $Nrxn1^{AS4-}$ p=0.0035, $Nrxn1^{AS4+}$ p<0.0001, $Nrxn2^{AS4-}$ p<0.0001, $Nrxn2^{AS4+}$ p<0.0001, $Nrxn3^{AS4-}$ p<0.0001, $Nrxn3^{AS4+}$ p<0.0001, *wt* SST vs ∆*SST* $Nrxn2^{AS4-}$ p=0.0251.

Figure S4

a

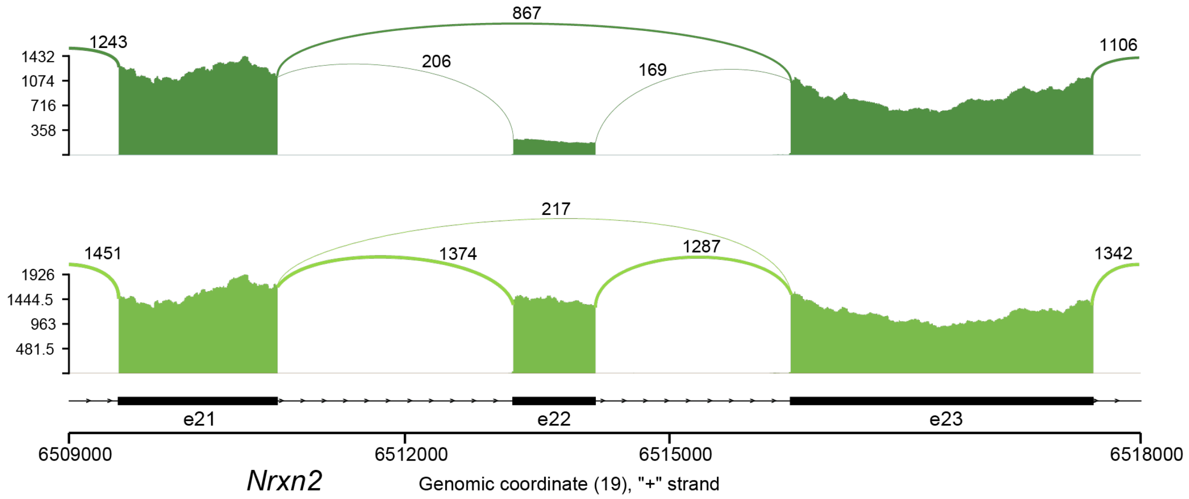

b

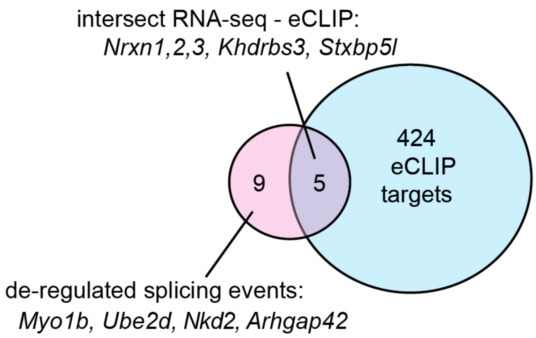

c

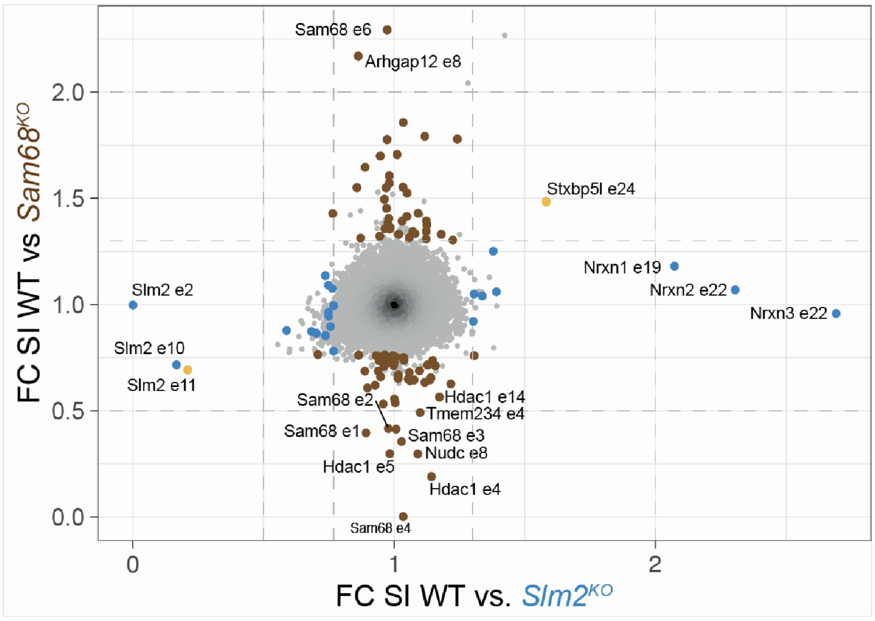

Figure S4. Alternative splice targets in *Slm2* conditional knock-out cells.

a, Representative sashimi plots illustrating read distribution and splice junctions on the *Nrxn2* gene at *AS4* for *wt* and ∆*Grik4* conditional mutant. Genomic coordinates and exon numbers are indicated below. Junction reads for exon-exon boundaries are noted and illustrated by line thickness. **b,** Venn diagram demonstrating the number of genes identified by eCLIP/IDR as bound (424), differentially alternatively spliced in *Slm2*^{KO} (9) or both bound and alternatively spliced (5). **c,** Correlation plot of the splicing index fold change (FC SI) in mouse hippocampus between WT and *Slm2* global knock-out (*Slm2*^{KO} [2]) and WT and Sam68 global knock-out (*Sam68*^{KO} [3]) for all detected exons (grey). Significantly differentially regulated exons (FC ± 30%, p-value 0.01) are marked in brown for *Sam68* and blue for *Slm2* mutants. Two exons, marked in yellow, are commonly de-regulated suggesting very little overlap in splicing regulation by SAM68 and SLM2 proteins.

Figure S5

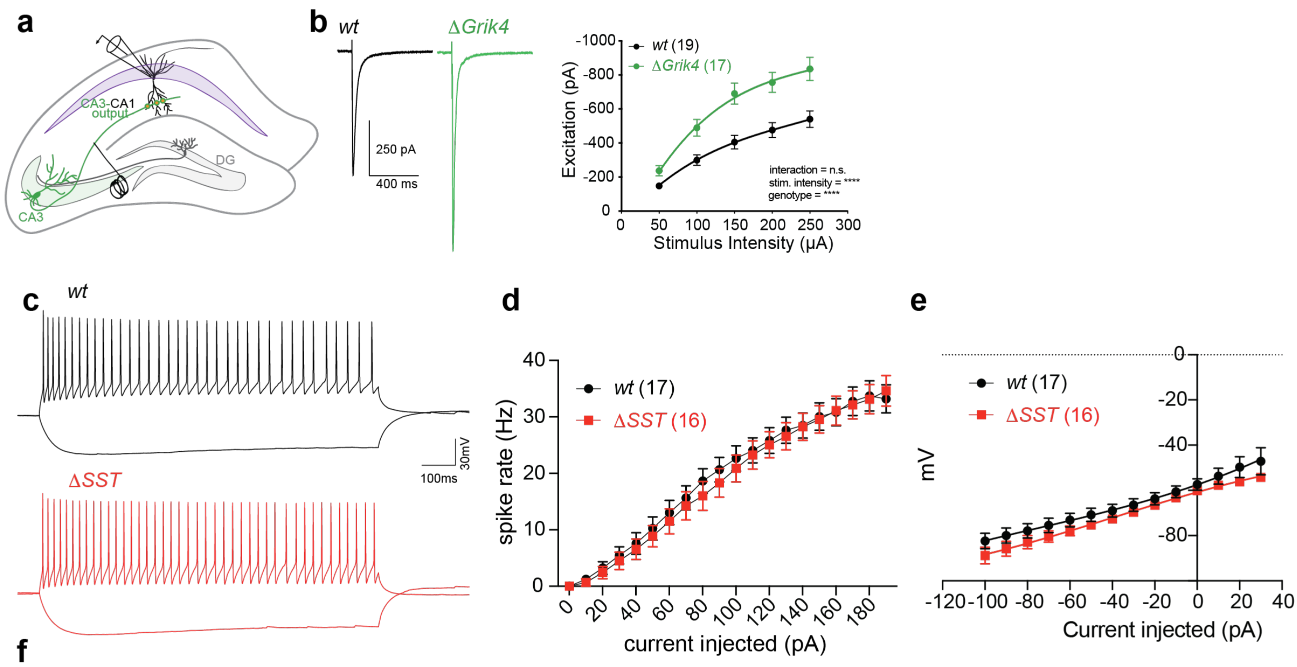

f

	WT	Slm2$^{\Delta SST}$	p-value	n (cells)
IR (MΩ)	622.9 ± 52.92	603.0 ± 46.64	0.7778	28 / 32
Capacitance (pF)	95.07 ± 7.498	101.0 ± 5.103	0.1116	28 / 32
RMP (mV)	-59.29 ± 1.964	-61.04 ± 1.250	0.4616	14 / 15
AP threshold (mV)	-38.31 ± 1.297	-38.57 ± 1.581	0.9027	14 / 15
AP amplitude (mV)	90.53 ± 3.277	93.41 ± 2.809	0.7130	14 / 15
AP Latency (ms)	83.86 ± 7.289	84.05 ± 5.358	0.4864	15 / 15
AP slope rise	283.9 ± 18.11	276.6 ± 14.64	0.4363	15 / 15
AP slope decay	116.0 ± 9.199	109.9 ± 6.326	0.2496	15 / 15
Rheobase (pA)	35.76 ± 7.923	42.27 ± 5.828	0.1612	17 / 15
Sag (mV)	-8.903 ± 1.817	-8.617 ± 1.084	0.8934	18 / 15

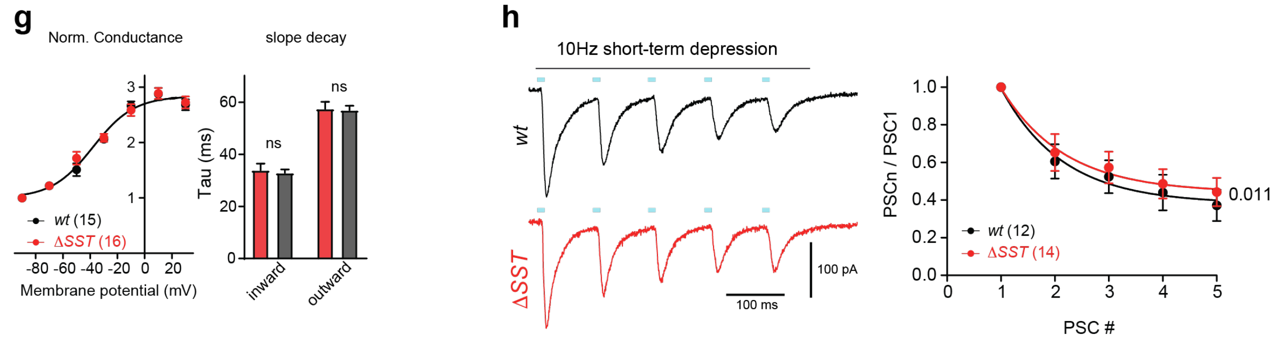

Figure S5. Electrophysiological analysis of *Slm2* conditional knock-out cells.

a, Experimental design for electrical stimulation of Schaffer collaterals and voltage clamp recordings in CA1 pyramidal cells in *wt* and ∆*Grik4* mutants. **b,** Representative traces of post-synaptic EPSCs *wt* (black) and ∆*Grik4* mutants (green). Electrically evoked EPSCs with various stimulation intensities in *wt* (n=19) and ∆*Grik4* mutants (n=17). Mean SD is displayed, two-way ANOVA was used for statistical analysis. **c,** Representative current clamp recordings to measure spike frequency of *wt* (black) and ∆*SST* (red) SST+ interneurons in s.o. Responses to a single 1s long -100pA or +150pA current injection. **d,** Frequency of action potential firing in response to increasing current injections. *wt* n= 17, ∆*SST* n=16 **e,** Analysis of changes in membrane potentials with increasing current in pA. The resting membrane potential is displayed at 0pA injection. *wt* n= 17, ∆*SST* n=16 **f,** Summary table of intrinsic electrophysiological properties of *wt* and ∆*SST* neurons. Mean ± SEM, p-values were determined by the corresponding t-tests based on assessment of normality distribution and standard deviation (see methods for details). **g,** left, a plot of normalized conductance versus membrane potential shows a clear voltage-dependence *wt* and ∆*SST* mutants. There was no genotype-dependent difference (p=0.60, Extra sum-of-squares F test). Right, analysis of decay times, weighted tau in milliseconds, of inward (at -90mV) and outward (at -10mV) currents showed no difference between genotypes (*wt* n=16 and ∆*SST* n=15) **h,** Example traces of IPSCs during repetitive stimulation at 10Hz, *wt* (black) and ∆*SST* (red). Group data of IPSCs normalized to the first peak. Mean ± SEM, *wt* n=14 and ∆*SST* n=12, Extra sum-of-squares F test for comparison of independent fits.

Figure S6

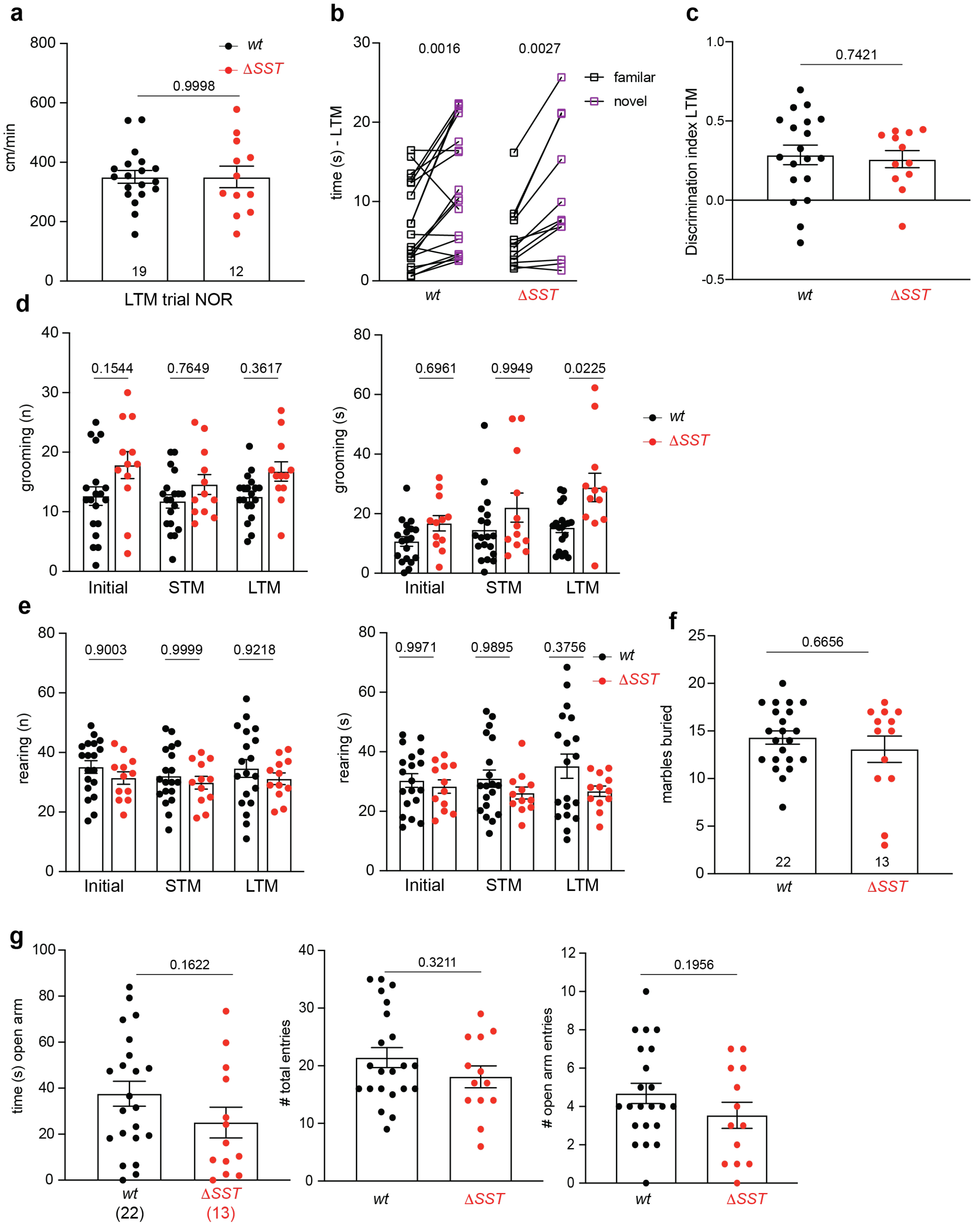

a
cm/min
wt
ΔSST
0.9998
19
12
LTM trial NOR
b
time (s) - LTM
0.0016
0.0027
familar
novel
wt
ΔSST
c
Discrimination index LTM
0.7421
wt
ΔSST
d
grooming (n)
0.1544
0.7649
0.3617
Initial
STM
LTM
grooming (s)
0.6961
0.9949
0.0225
wt
ΔSST
e
rearing (n)
0.9003
0.9999
0.9218
rearing (s)
0.9971
0.9895
0.3756
f
marbles buried
0.6656
22
13
g
time (s) open arm
0.1622
(22)
(13)
total entries
0.3211
open arm entries
0.1956

Figure S6. Behavioral assessments in *Slm2$^{\Delta SST}$ mice*

a, Quantification of velocity (cm/min) of mice during the long-term memory (LTM) test phases of the novel object recognition (NOR) task. Animal numbers for each task are indicated, Mean ± SEM, Unpaired t-test. **b,c**, Interaction time (in seconds) that mice spend with either a familiar (black) or novel (purple) object during a 5-min long-term memory trial (paired t-test) and discrimination index (unpaired t-test with Welch's correction) are displayed. Mean ± SEM, *wt* n=19 and Δ*SST* n=12. **d,** Quantification of the number (left) and duration (right) grooming events of mice during the Open Field and phases of the NOR task. Mean ± SEM, One-Way ANOVA with Tukey's multiple comparisons test. **e,** Quantification of the number (left) and duration (right) rearing events of mice during the Open Field and phases of the NOR task. Mean ± SEM, One-Way ANOVA with Tukey's multiple comparisons test. **f,** Number of marbles buried when mice are placed in a novel homecage including 20 black marbles for 30min. Mean ± SEM, Mann Whitney t-test. *wt* n=22 and Δ*SST* n=13. **g**, Analysis of the amount of time mice spend in an open arm of the elevated plus maze during a 5min trial (left), their number of entries into either the open or closed arm (middle) and number of entries into the open arm (right). Mean ± SEM, unpaired t-test. *wt* n=22 and Δ*SST* n=13.

References Cited in Supplement

1. Furlanis, E., Traunmuller, L., Fucile, G., and Scheiffele, P. (2019). Landscape of ribosome-engaged transcript isoforms reveals extensive neuronal-cell-class-specific alternative splicing programs. Nature neuroscience *22*, 1709-1717. 10.1038/s41593-019-0465-5.
2. Traunmüller, L., Gomez, A.M., Nguyen, T.-M., and Scheiffele, P. (2016). Control of neuronal synapse specification by highly dedicated alternative splicing program. Science (New York, N.Y *352*, 982-986.
3. Witte, H., Schreiner, D., and Scheiffele, P. (2019). A Sam68-dependent alternative splicing program shapes postsynaptic protein complexes. Eur J Neurosci *49*, 1436-1453. 10.1111/ejn.14332.
